# PEDF and its roles in physiological and pathological conditions: implication in diabetic and hypoxia-induced angiogenic diseases

**DOI:** 10.1042/CS20130463

**Published:** 2015-03-19

**Authors:** Xuemin He, Rui Cheng, Siribhinya Benyajati, Jian-xing Ma

**Affiliations:** *Department of Physiology, The University of Oklahoma Health Sciences Center, Oklahoma City, OK, USA.

**Keywords:** angiogenesis, diabetes, hypoxia, pigment epithelium-derived factor (PEDF), PEDF receptor, serpin, tissue distribution, AGE, advanced glycation end-product, ATGL, adipose triglyceride lipase, ATRA, all-*trans* retinoic acid, bFGF, basic fibroblast growth factor, c-FLIP, cellular FLICE-like inhibitory protein, CK2, casein kinase 2, DR, diabetic retinopathy, E, embryonic day, ERK, extracellular-signal-regulated kinase, ER, oestrogen receptor, FasL, Fas ligand, Flt-1, FMS-like tyrosine kinase 1, HIF-1, hypoxia-inducible factor 1, HUVEC, human umbilical vein endothelial cell, IκB, inhibitor of κB kinase, IGF, insulin-like growth factor, JNK, c-Jun N-terminal kinases, KDR, kinase insert domain receptor, LR, laminin receptor, LRP, low-density lipoprotein receptor-related protein, MAPK, mitogen-activated protein kinase, MEK5, MAPK/ERK kinase 5, MMP, matrix metalloproteinase, NFAT, nuclear factor of activated T-cells, NFATc2, nuclear factor of activated T-cells cytoplasmic 2, NF-κB, nuclear factor κB, OIR, oxygen-induced retinopathy, PAI-1, plaminogen activator inhibitor-1, PEDF, pigment epithelium-derived factor, PEDF-R, PEDF receptor, PEDF-tg, transgenic overexpression of PEDF, PDGF, platelet-derived growth factor, PKA, protein kinase A, PPAR-γ, peroxisome proliferator-activated receptor γ, RAR, retinoic acid receptor, RARE, retinoic acid-response element, RCL, reactive centre loop, RPE, retinal pigment epithelial, RXR, retinoid X receptor, serpin, serine proteinase inhibitor, VEGF, vascular endothelial growth factor

## Abstract

Pigment epithelium-derived factor (PEDF) is a broadly expressed multifunctional member of the serine proteinase inhibitor (serpin) family. This widely studied protein plays critical roles in many physiological and pathophysiological processes, including neuroprotection, angiogenesis, fibrogenesis and inflammation. The present review summarizes the temporal and spatial distribution patterns of PEDF in a variety of developing and adult organs, and discusses its functions in maintaining physiological homoeostasis. The major focus of the present review is to discuss the implication of PEDF in diabetic and hypoxia-induced angiogenesis, and the pathways mediating PEDF's effects under these conditions. Furthermore, the regulatory mechanisms of PEDF expression, function and degradation are also reviewed. Finally, the therapeutic potential of PEDF as an anti-angiogenic drug is briefly summarized.

## INTRODUCTION

Pigment epithelium-derived factor (PEDF), also known as early population doubling level cDNA-1 [1,2], was originally isolated from the conditioned medium of cultured human fetal retinal pigment epithelial cells [3] and found to possess neuronal differentiation properties [4]. It is a highly conserved glycoprotein in mammals [5] and possesses a reactive centre loop (RCL) [5] that is a common structural characteristic of the serine proteinase inhibitor (serpin) family. Cleavage within the RCL by chymotrypsin does not impair PEDF's functions [6], suggesting that PEDF is a non-inhibitory serpin [6,7]. A decade after PEDF's identification, Dawson et al. [8] demonstrated PEDF as a potent endogenous anti-angiogenic factor. This opened a new era for the exploration of PEDF's functions in angiogenic diseases, especially in diabetes [9–11] and cancer [12–14]. PEDF levels were found to decline in angiogenic tissues/organs, such as the vitreous, aqueous humors and retinas from patients with proliferative diabetic retinopathy (DR) and in tumours from cancer patients [10,15–23]. Interestingly, circulating PEDF levels in proliferative DR patients are increased relative to diabetic patients without proliferative DR or non-diabetic controls [24–27]; this observation possibly reflects a systemic compensatory response to angiogenesis in proliferative DR. Thus restoration of PEDF levels within angiogenic sites could be a promising strategy for the treatment of angiogenesis-related diseases.

The PEDF protein plays fundamental roles in organogenesis [12,28–30] and homoeostatic maintenance of adult tissues/organs [12,14,31–33]. Defects or deficiencies of PEDF expression are closely associated with progression of angiogenic diseases [10,15–18,25,27,34,35]. The present review summarizes the temporal and spatial distribution of PEDF in multiple organs during developmental stages and adulthood, and discusses its implication in diabetic and hypoxia-induced angiogenic diseases. In addition, we review PEDF's anti-angiogenic mechanisms under these two types of acquired angiogenic conditions. Finally, the regulatory mechanisms of PEDF expression, function and degradation are reviewed, and the therapeutic potential of PEDF in angiogenic diseases is briefly discussed.

## TISSUE DISTRIBUTION AND PHYSIOLOGICAL FUNCTIONS OF PEDF

The mRNA encoding the human PEDF (*SERPINF1* mRNA) is expressed in most organs, including the liver, adipose tissue, eye, heart, kidney, ovary, testis, spleen, skeletal muscle, brain and bone [36]. Deficient or defective expression of PEDF leads to abnormal organ development. It was reported that human patients with undetectable circulating PEDF develop osteogenesis imperfecta type VI [28,37]. In mouse, global PEDF deficiency does not affect viability and fertility [12], but leads to pancreatic and prostatic hyperplasia [12], hepatic steatosis [31] and bone defects similar to human patients with osteogenesis imperfecta type VI [29], indicating that PEDF participates in important physiological events in both humans and animals.

### PEDF in the liver

*SERPINF1* mRNA is highly expressed in human fetal and adult livers [36]. In normal human and mouse livers, hepatocytes are the predominant PEDF-expressing cells [38,39]. In mouse embryonic livers, PEDF protein is detected as early as embryonic day (E) 12.5, with its expression continuously increasing during organogenesis and remaining at high levels in adult livers [39]. PEDF regulates lipid metabolism and maintains physiological homoeostasis in mouse livers. Significant accumulation of neutral lipid and triglyceride is observed in hepatocytes of 1-month-old PEDF-deficient mice, and is continuously increased with age, whereas restoration of PEDF decreases triglyceride content in PEDF-deficient hepatocytes [31]. The regulatory effect of PEDF on lipid metabolism is mediated by adipose triglyceride lipase (ATGL) [40]. PEDF activates ATGL to promote adipose lipolysis, which may contribute to insulin resistance in obese subjects [41] as ATGL-deficient mice do not develop PEDF-induced insulin resistance [40]. PEDF deficiency in the liver also results in hepatic steatosis as observed in adult PEDF-deficient mice even under normal feeding conditions [31]. When fed an alcohol-containing liquid diet, enhanced expression of the fibrotic marker α-smooth muscle actin is detected in the hepatic perisinusoidal space of PEDF-deficient mice compared with wild-type controls [38]. Consistently, in the livers from patients and animal models with hepatic steatosis, PEDF expression is dramatically down-regulated [38,42]. Overexpression of PEDF reverses liver fibrosis [42]. Clinical studies indicate a positive correlation between circulating PEDF levels and insulin resistance in patients who are morbidly obese [43] or diabetic [44–47]. It is known that insulin resistance leads to compromised hepatic glycogenesis and gluconeogenesis in both diabetic patients [48,49] and diabetic animals [50]; however, it is currently unknown whether the insulin resistance-induced aberrant gluconeogenesis and glycogenesis in the liver is due to elevated PEDF levels in obesity and diabetes.

### PEDF in the pancreas

The *SERPINF1* mRNA is weakly detected in the human pancreas [36]. Higher levels of human PEDF protein are detected in centroacinar cells and islet cells in comparison with most acinar cells and ductal cells [51]. PEDF regulates pancreatic vasculature development, and PEDF deficiency in the pancreas leads to a 2.6-fold increase in microvessel density and more dilated and thicker walled blood vessels [12]. In addition, PEDF deficiency can result in atypical hyperplastic phenotypes in the pancreas, such as enlarged pancreas, more exocrine glands, less differentiated acinar epithelial cells, and increased proliferating epithelial cells [12]. Furthermore, PEDF deficiency makes the pancreas more prone to fibrosis as PEDF-deficient pancreases display significantly increased basal transcription levels of collagen αI, transforming growth factor β1 and platelet-derived growth factor (PDGF) [32]. Enhanced expression of α-smooth muscle actin is also detected in both of the pancreatic vessels and ducts of PEDF-deficient mice [12,32].

The EL-Kras^G12D^ mouse is a transgenic model that spontaneously develops pancreatic lesions and non-invasive cystic papillary neoplasms. At the age of 6–12 months, EL-Kras^G12D^/PEDF-deficient mice develop more severe acinar ductal metaplasia and cystic papillary neoplasm compared with age-matched EL-Kras^G12D^ mice. Moreover, relative to aged controls, aged EL-Kras^G12D^/PEDF-deficient mice exhibit more severe pathological changes such as increased ductal metaplasia, augmented papillary neoplasm frequency, enlarged adipocytes, elevated tubular complexes and remarkable multifocal dysplastic changes [52]. Although PEDF is known to affect pancreatic vasculature, morphology and function, PEDF's expression and roles in the pancreas under diabetic conditions remain to be elucidated.

### PEDF in the lung

The *SERPINF1* mRNA is modestly detected in the human fetal lung, and persists throughout adulthood [36]. PEDF protein is expressed in human pulmonary epithelia, pulmonary fibroblasts/myofibroblasts, alveolar interstitium and bronchoalveolar lavage fluid [53]. PEDF has been suggested to be an important regulator of pulmonary angiogenesis and fibrosis. Lung-derived endothelial cells from PEDF-deficient mice exhibit enhanced migratory capacity and adherent ability compared with cells from wild-type mice, which may be ascribed to decreased cell surface integrins and increased vascular endothelial growth factor (VEGF) secretion [33]. Moreover, both arteriole and venule walls in PEDF-deficient lungs are thicker [33].

Wild-type mice exposed to hyperoxia from postnatal day 5–13 develop impaired alveolarization, which is accompanied by increased PEDF levels mainly in alveolar epithelia [54]. However, hyperoxia-compromised alveolarization is not detected in aged-matched PEDF-deficient mice [54], further implying that increased PEDF levels are the primary causative factor for reduced alveolarization. In idiopathic pulmonary fibrosis that is recently believed to be characterized by impaired angiogenesis instead of enhanced angiogenesis [55], pulmonary PEDF levels are dramatically increased and inversely correlated with VEGF levels and pulmonary microvascular density [53]. It is believed that increased PEDF levels prohibit pulmonary angiogenesis, thus exacerbating idiopathic pulmonary fibrosis [53].

### PEDF in the bone

*SERPINF1* mRNA is highly expressed in adult human bone marrow [36]. In mice, high levels of PEDF protein are detected in the epiphyseal cartilage (resting, proliferating and upper hypertrophic chondrocytes) and the periosteum (active osteoblasts and chondrocytes), suggesting that PEDF is spatially expressed in areas of endochondral ossification and bone remodelling [56]. Interestingly, PEDF protein is distributed in similar spatial patterns in newborn mice and 5- and 9-week-old mice [56]. The principal cell types that express and secrete PEDF are osteoblasts [57] and chondrocytes [56]. PEDF affects bone formation and resorption by the regulation of osteoblastic and osteoclastic differentiation [58–60]. PEDF promotes osteoblastic differentiation by inducing the expression of osteoblastic genes [58,60]. On the other hand, PEDF impedes osteoclastic differentiation by up-regulating osteoclast-repressive genes [59]. PEDF also affects bone mineralization [29,58,60]. PEDF treatment in human mesenchymal stem cells increases alkaline phosphatase activity that is critical in the mineralization process [60]. Interestingly, mesenchymal stem cells isolated from PEDF-deficient mice show reduced alkaline phosphatase expression [61]. Moreover, both PEDF protein and PEDF-derived peptides (residues 40–64, residues 78–102 and residues 90–114 corresponding to the full-length immature human PEDF sequence) enhance bone mineralization [58,62].

In patients with osteogenesis imperfecta type VI, a disease characterized by low bone mass and reduced bone strength, a homozygous frame-shift mutation in exon 4 or a termination mutation in exon 8 in the *SERPINF1* gene is detected, resulting in undetectable levels of PEDF protein in the circulation [28]. Clinically, the circulating PEDF level is a critical biomarker for the screening of this disease [63]. Bogan et al. [29] reported that PEDF-deficient mice exhibited symptoms of osteogenesis imperfecta type VI patients with significant increases in osteoid maturation time, unmineralized bone matrix, mineral/matrix ratio and bone fragility. Notably, patients with heterozygous null mutated *SERPINF1* gene possess normal and functional bones, although their circulating PEDF is significantly lower than that of control patients with a fully functional *SERPINF1* gene [64].

However, circulating PEDF levels should be interpreted with caution, and not used as the sole biomarker for this disease. Recently, an atypical case of osteogenesis imperfecta type VI was reported in which the patient had normal serum PEDF levels and correct *SERPINF1* gene sequence, but a heterozygous single point mutation in the *IFITM5* gene [65]. The single point mutation of *IFITM5* does not affect its own transcript, instead, it reduces the expression and secretion of PEDF in fibroblasts and osteoblasts isolated from the patient [65]. Why this mutation in *IFITM5* affects PEDF expression and secretion and how it leads to osteogenesis imperfecta type VI remains unknown.

The Wnt/β-catenin pathway plays a pivotal role in bone formation and homoeostasis [66]. In bone formation, PEDF and Wnt/β-catenin play similar roles: increased PEDF or activation of Wnt/β-catenin signalling leads to high bone mass, whereas decreased PEDF or attenuation of the Wnt/β-catenin signalling results in low bone mass [29,66]. A recent study reported PEDF as an agonist of the Wnt/β-catenin pathway in human mesenchymal stem cells [61]. Another study also reported that treatment of PEDF in osteocytes induced phosphorylation of glycogen synthase kinase 3β (GSK-3β) and total β-catenin levels [58]. Our group and another group demonstrated that PEDF is an inhibitor of the Wnt/β-catenin pathway in an angiogenic eye model [67] and a wound-healing model [68]. Discrepancy between these studies is noted, yet conclusive explanation is unavailable at this moment and further studies are needed. Notably, in bone-derived cells, whether PEDF's stimulative effect on the Wnt/β-catenin pathway is mediated through low-density lipoprotein receptor-related protein 6 (LRP6) is not established. In addition, PEDF strongly inhibits the expression of sclerostin, an inhibitor of Wnt/β-catenin signalling [69], which might contribute to the activation of Wnt/β-catenin signalling observed in osteocytes [58].

### PEDF in the kidney

*SERPINF1* mRNA is moderately expressed in human fetal kidneys, and its level declines in adult human kidneys [36]. In human kidneys, PEDF protein is predominantly detected in tubules and with less intensity in glomeruli [70]. In mouse kidneys, PEDF protein localizes in the renal vasculature, interstitial spaces, glomeruli and tubules [71]. In rat kidneys, PEDF protein levels continuously increase with age [72]. In neonatal rats, PEDF protein is restricted to glomerular and capillary mesenchyme and endothelial cells of the nephrogenic zones [72]. By postnatal day 7, PEDF is also detected in the Bowman's capsules of maturing glomeruli locating at the inner cortical region [72]. The first detection of PEDF in podocytes close to the innermost region is at postnatal day 14 [72]. In adult rats, high levels of PEDF are predominantly detected in glomerular podocytes and endothelial cells, and vascular endothelial cells [72,73]. It is noteworthy that PEDF protein expression persists in all types of blood vessels in the kidneys throughout all fetal stages and adulthood. The significance of PEDF in renal vascular development has been well demonstrated, as renal microvessel density of PEDF-deficient mice is greatly increased compared with that in wild-type controls [12,14]. In a diabetic rat model, renal PEDF levels are significantly decreased [73], although PEDF levels in the kidneys of diabetic patients have not been measured. Clinical studies have shown that PEDF levels are closely correlated with vascular dysfunction-associated renal diseases. In patients with chronic kidney disease, plasma PEDF levels are significantly increased compared with those of patients without chronic kidney disease [74]. Moreover, in both Type 1 and Type 2 diabetes, serum PEDF levels correlate positively with serum creatinine concentrations [44,45,47], and inversely with glomerular filtration rate [44,47,75]. It is still unclear what roles PEDF plays in these renal diseases and whether its roles are related to its effects in the vasculature.

### PEDF in the eye

The PEDF protein is detected in the human eye from early embryonic stages [76]. In very early stage of human fetal development, PEDF is restricted to the granules of retinal pigment epithelial (RPE) cells, the ganglion cell layer and sporadic cells in the neuroblastic layer [76]. In later human fetal stages, PEDF protein is found in RPE cells, corneal epithelia and endothelia, ciliary body non-pigmented epithelia and muscle, horizontal cells in the outer part of the inner nuclear layer, differentiating photoreceptors and apical cytoplasm of differentiating cones [76]. In adult human retinas, PEDF protein localizes in rods and cones, the inner nuclear layer, ganglion cell layer and inner plexiform layer. The choroid, corneal epithelia and endothelia, ciliary body non-pigmented epithelia and muscle, RPE cells and photoreceptors are also PEDF-positive [76]. In the macula of monkeys at all ages, the *SERPINF1* mRNA is detected in the ganglion cell layer and RPE cells, with the highest level present at the fovea [77]. Abundant PEDF protein is also found in the monkey interphotoreceptor matrix surrounding rod and cone outer segments, with lower levels detected in the vitreous and aqueous humors due to the polarized and directional secretion of PEDF towards the neural retina by RPE cells [78]. A number of studies have reported very high levels of PEDF expression in RPE cells [76–78], and polarized PEDF secretion is closely associated with the polarization and maturation of RPE cells [78–80]. As high levels of PEDF are found in the interphotoreceptor matrix, vitreous and aqueous humors in species such as the cow [81–83] and human [76,83], purification from these sources was a common way to obtain PEDF before the eukaryotic expression system of PEDF was established. In mouse embryonic stages, PEDF protein is firstly detected at E14.5 in the inner plexiform layer and RPE cells, followed by detection at E18.5 in the ganglion cell layer, inner nuclear layer, ciliary body and choroid [84]. In rats, *Serpinf1* mRNA is localized in the lens epithelia, RPE cells, ciliary epithelia, retinal ganglion cells and sporadic cells of the inner nuclear layer, and PEDF protein is identified in RPE cells, corneal epithelia and endothelia, ciliary epithelia, the nerve fibre layer, ganglion cell layer and inner and outer plexiform [85].

PEDF affects retinal vasculature via its anti-angiogenic properties. Compared with wild-type controls, the retinas of PEDF-deficient mice display the following changes: a faster vascular expansion rate from postnatal day 3 to 7, significantly increased deep vascular plexi at postnatal day 10 and a slightly increased endothelial cell/pericyte ratio at postnatal day 21 [30]. Adult PEDF-deficient mice at the age of 3 months also exhibit a 5.2-fold increase of retinal microvessel density and malpositioned vessels [12]. In addition, PEDF deficiency results in more severe vessel obliteration in the oxygen-induced retinopathy (OIR) model [30]. In a mouse model of DR, loss of PEDF results in a 2-fold increase of acellular capillaries [86]. In contrast, genetic overexpression of PEDF in mice significantly suppresses retinal neovascularization in the OIR model [87]. Similarly, adeno-associated virus overexpressing PEDF dramatically represses retinal neovascularization in an insulin-like growth factor 1 (IGF-1) transgenic mouse model [88]. In addition to the retina, PEDF and PEDF-derived peptides have also been demonstrated to strongly inhibit choroidal neovascularization [87,89–91] and corneal neovascularization [8,92–94]. Taken together, these studies indicate that PEDF is indeed a potent endogenous angiogenic inhibitor in the eye.

Lack of PEDF has subtle impacts on mouse retinal development, differentiation and function under normal conditions [30]. Yet PEDF plays an important supporting role in promoting retinal differentiation and maintaining retinal function. PEDF exhibits a pro-cone trophic action in a chicken rosetted retinal spheroids model by inducing cone opsin expression and decreasing rod numbers [95]. PEDF also promotes the differentiation and maturation of RPE cells [96]. In cultured heterogenic cell clusters from rat retinas, the RPE cell population is dramatically increased by PEDF accompanied with the following changes: increase in size, loss of vacuolization, acquisition of a more epithelium-like appearance, enhanced cell adhesion property and increased mature pigment granules [96]. Moreover, overexpression of PEDF causes bone marrow stromal cells to differentiate to RPE cells [97]. In addition, the neurotrophic function of PEDF promotes the survival of retinal neurons [98,99], retinal progenitor cells [80], RPE cells [100] and photoreceptors [98,100–103]. Notably, the cytoprotective effect of PEDF in retinal cells is reported to be mediated via the PEDF receptor (PEDF-R) [104]. Thus PEDF is indeed a demonstrable retinal protective factor.

## IMPLICATION OF PEDF IN DIABETIC AND HYPOXIA-INDUCED ANGIOGENIC DISEASES

Angiogenesis is involved in many physiological and pathological processes, and is stimulated by angiogenic factors [105]. Angiogenic stages are composed of endothelial activation, sprouting, regression and maturation [106]. PEDF exerts its anti-angiogenic effects primarily by targeting endothelial cells. PEDF's functions in tumour angiogenesis have been reviewed by Hoshina et al. [107], Manolo et al. [108] and Becerra and Notario [109]. Rychlic et al. [110] and Liu et al. [111] have reviewed PEDF's roles in cardiovascular angiogenesis. In the present review, we focus on PEDF's functions in two major types of acquired angiogenesis: diabetic angiogenesis and hypoxia-induced angiogenesis.

### PEDF-Rs in angiogenic regulation

PEDF-R (also known as ATGL/desnutrin/iPLA2-ζ/TTS2.2), encoded by the *PNPLA2* gene in humans, is the first identified receptor for PEDF [112]. PEDF-R is expressed in human ocular tissues (fetal and adult RPE layers and retinas) [112], human Y-79 cells [112,113], RPE cells (including ARPE-19 and hTERT) [112,114], 661W (a mouse photoreceptor cell line) [115], human adipose tissue and 3T3-L1 cell line [112], bovine retina and RPE cells [114,116], rat R28 (a photoreceptor precursor cell line) [112], and a RGC-derived cell line [112,114,117]. PEDF-R mediates multiple activities of PEDF [40,104,118–123]. There is no direct evidence showing that PEDF's anti-angiogenic activities are mediated through PEDF-R in endothelial cells; however, it has been demonstrated that PEDF induces the expression of apoptotic Fas ligand (FasL) by regulating nuclear factor κB (NF-κB) in endothelial cells [124], and that the regulation of NF-κB by PEDF is PEDF-R-dependent [118]; thus it may be possible that PEDF exerts its anti-angiogenic activities through PEDF-R in endothelial cells.

Laminin receptor (LR) is another receptor identified to mediate PEDF's anti-angiogenic effects [125]. Knockdown of LR attenuates PEDF-induced endothelial cell apoptosis and migration [125]. A triple phosphomimetic mutant of PEDF, possessing more potent anti-proliferative, anti-migratory and pro-apoptotic effects in endothelial cells, exhibits higher binding affinity to LR, but not to PEDF-R compared with wild-type PEDF, suggesting that enhanced anti-angiogenic properties of the PEDF triple phosphomimetic mutant are mediated through LR [126].

F_1_-ATP synthase was also found to convey PEDF's anti-angiogenic activities. In endothelial cells, PEDF binds to the β-subunit of the F_1_-ATP synthase in a specific, reversible and saturable manner (*K*_d_=1.51 nM in real-time surface plasmon resonance assay, *K*_d_=3.04–4.97 nM in bovine retinal endothelial cells), resulting in a reduced F_1_-ATP synthesis activity and subsequently lower ATP levels, which impedes the energy supply for angiogenic events in endothelial cells [127].

Some serpins are known to bind to LRPs: SERPINE1 [128], SERPINE2 [129] and SERPINA1 [130] bind to LRP1; SERPINE1 [128] and SERPINA3 [130] bind to LRP2; and SERPINA3K binds to LRP6 [131]. Our previous study also demonstrated that PEDF binds to LRP6 [67], a co-receptor of the Wnt/β-catenin pathway that plays important pathogenic roles in retinal inflammation, neovascularization and vascular leakage in angiogenic eye models [67,87,131–133]. PEDF-deficient OIR retinas show enhanced activation of the Wnt/β-catenin pathway compared with wild-type OIR retinas [67], whereas transgenic overexpression of PEDF in mouse attenuates the Wnt/β-catenin signalling and neovascularization in the OIR and laser-induced choroidal neovascularization models [87].

An additional group of atypical non-canonical ‘receptors’, also known as extracellular proteins, has been reported to affect PEDF's angiostatic functions. PEDF binds to collagen I, which might modulate the integrin–collagen I interaction, thus affecting endothelial cell adhesion and docking [134,135]. In addition, PEDF binds to collagen II [134], collagen III [136] and glycoaminoglycans [137–140], which are also very likely to facilitate PEDF's anti-angiogenic functions.

### Role of PEDF in diabetic angiogenesis

DR and diabetic nephropathy are diabetes-related angiogenic diseases. Proliferative DR is characterized by increased formation of immature vessels in the retina and vitreous, which can ultimately lead to intra-retinal or pre-retinal haemorrhage [141]. In the kidneys from 50-day-old diabetic rats, average capillary areas per glomerulus, capillary length and capillary numbers are significantly increased [142]. Diabetic angiogenesis is induced by factors including hyperglycemia, hypoxia, imbalanced redox states, elevated non-enzymatic glycosylation and nitration, etc. [141]. Notably, compared with either non-diabetic individuals or diabetic patients with non-proliferative DR, PEDF levels are markedly decreased in the vitreous, retinas and aqueous humors of diabetic patients with proliferative DR [10,15,16,18,143–149]. PEDF levels are also decreased in the kidneys from Type 1 diabetic mice and rats [47,71,73,150]. In contrast, PEDF levels in the circulation are significantly increased in diabetic patients relative to non-diabetic patients [24,25,27,44,45,47,151], and positively correlated with the severity of diabetic complications [24,27,44,47,151].

PEDF counteracts angiogenesis in both proliferative DR [152–154] and diabetic nephropathy [155]. Compared with other endogenous anti-angiogenic factors such as thrombospondin, endostatin and angiostatin, PEDF is more effective in inhibiting endothelial cell migration [8]. Activation of the p38 mitogen-activated protein kinase (p38 MAPK) mediates PEDF's anti-migratory effect in bovine aorta endothelia cells [126]. The receptor that transmits PEDF's effect to p38 MAPK was reported to be LR [126]. Matrix metalloproteinases 2/9 (MMP-2/9) promote angiogenesis by degrading the extracellular matrix, thus mobilizing endothelial cells [156]. PEDF down-regulates the activities of MMP-2/9 in the aqueous humor of a proliferative DR model [88].

In addition, PEDF inhibits the proliferation of endothelial cells by regulating the MAPK/extracellular-signal-regulated kinase (ERK) pathways [126,153,157–159]. The Wnt/β-catenin pathway is activated in diabetic angiogenesis [160] and promotes endothelial cell migration and proliferation via its targets VEGF [161] and MMPs [162–164]. As demonstrated by ligand-binding assays and co-immunoprecipitation, PEDF binds to LRP6, a co-receptor in the Wnt/β-catenin pathway, and suppresses Wnt signalling in ARPE-19 and Müller cells [67].

Quenching oxidative stress is another mechanism by which PEDF inhibits diabetic angiogenesis. PEDF suppresses nicotinamide adenine dinucleotide phosphate oxidase activity in the retinas of diabetic rats [152] and rats with retinal hyper-permeability induced by advanced glycation end-products (AGEs) [165]. Similarly, in *ex vivo* endothelial cell cultures, PEDF directly suppresses reactive oxygen species generation by inhibiting NADPH oxidase activity elicited by AGEs [165,166], tumour necrosis factor α [167] and angiotensin II [168]. Moreover, in bovine retinal endothelial cells, PEDF activates peroxisome-proliferator-activated receptor γ (PPAR-γ), which then up-regulates uncoupling protein 2 and subsequently decreases mitochondria-derived reactive oxygen species [169].

In addition to endothelial cells, pericyte loss has been well established to contribute to the angiogenic progression in DR [170]. PEDF protects pericytes against apoptosis induced by high glucose, H_2_O_2_, AGEs and oxidized low-density lipoprotein through its antioxidant and anti-inflammatory activities [171–173]. In addition, survival and proliferation of pericytes require endothelium-derived PDGF-BB [174]. PEDF promotes the proliferation of pericytes via up-regulating the expression of PDGF-BB [175].

### Role of PEDF in hypoxia-induced angiogenesis

In hypoxic disease states, the activation of hypoxia-inducible factor 1 (HIF-1) precedes the occurrence of angiogenesis. Hypoxia stabilizes HIF-1α to form a heterodimer with HIF-1β [176], which then activates the transcription of pro-angiogenic genes [177,178]. OIR is a widely used ischaemia-induced retinal angiogenesis model [179]. In OIR mouse retinas, HIF-1 and VEGF are up-regulated [180] and trigger the occurrence and progression of retinal angiogenesis that peaks between postnatal day 17–21 [179]. In contrast, PEDF is down-regulated in the choroid and RPE cells of this model from postnatal day 13–17 [181]. Similarly, in the retinas of OIR rats, PEDF levels are decreased with its lowest levels detected at postnatal day 16, which is coincidental with the peak expression of VEGF in the retinas of the same model [11]. To provide *in vivo* evidence of PEDF's anti-angiogenic activities in the OIR model, we generated PEDF transgenic (PEDF-tg) mice that overexpressed PEDF. Compared with wild-type OIR retinas, PEDF-tg OIR retinas display a significant reduction in retinal neovasculature [87]. In contrast, PEDF-deficient OIR retinas exhibit more prominent VEGF overexpression [30,67] and more severe angiogenesis relative to wild-type OIR retinas [30]. Laser-induced choroid neovascularization is another hypoxia-triggered angiogenic model [182]. Our group and other groups found that overexpression or delivery of PEDF or PEDF-derived peptide dramatically suppressed choroidal neovascularization [87,90,91,183,184].

PEDF suppresses hypoxia-induced angiogenesis by either directly targeting HIF-1 or regulating the expression or the signalling cascades of HIF-1's target genes. It is reported that PEDF blocks HIF-1 nuclear translocation and represses VEGF promoter activity under hypoxic conditions in retinal capillary endothelial cells [153]. However, the mechanism by which PEDF inhibits HIF-1 nuclear translocation remains unknown. Regulatory effects of PEDF on the expression of HIF-1 target genes and their signalling are summarized below.

*VEGF* is a target gene of HIF-1, with a hypoxia-response element located at its promoter region [185,186]. Our group and others demonstrated that PEDF decreased the expression of hypoxia-induced VEGF in retinal capillary endothelial cells, Müller cells and an angiogenic eye model [93,153]. VEGF/kinase insert domain receptor (KDR) is a crucial angiogenic pathway, and PEDF was reported to disrupt this pathway. Our previous study has shown that PEDF competes with VEGF for binding to KDR in retinal capillary endothelial cells, muting the angiogenic events of the VEGF/KDR pathway [153]. In addition, VEGF/FMS-like tyrosine kinase 1 (Flt-1) signalling was demonstrated to be essential for the survival of human dermal microvascular endothelial cells [187]. PEDF treatment activates γ-secretase in bovine retinal microvascular endothelial cells, which then triggers the cleavage of Flt-1 and mutes the phosphorylation of Flt-1 [188], leading to suppressed VEGF/Flt-1 signalling which is required for the viability [187] and tube formation ability of endothelial cells [188]. In addition, increased concentrations of the extracellular domain of Flt-1 trap and sequester VEGF in the extracellular matrix [188,189], resulting in less available VEGF and subsequent attenuated VEGF/KDR signalling.

Basic fibroblast growth factor (bFGF) is a potent angiogenic factor regulated by HIF-1 [190,191]. bFGF activates nuclear factor of activated T-cells (NFAT), an angiogenic transcription factor, to promote angiogenic events in human microvascular endothelial cells and human umbilical vein endothelial cells (HUVECs) [192]. In response to PEDF treatment, the association between c-Jun N-terminal kinase 2 (JNK-2) and NFAT cytoplasmic 2 (NFATc2) is significantly increased in endothelial cells, which leads to elevated cytoplasmic retention of NFATc2 and decreased nuclear levels of NFAT to promote angiogenesis [192]. In addition, bFGF induces bovine aorta endothelial cell migration, whereas PEDF treatment activates p38 to counteract bFGF-stimulated endothelial cell migration [126].

MMP-2 expression is modulated by HIF-1 in human somatic endothelial cells and HUVECs [193–195]. In addition, hypoxia affects the activation [196,197] and expression [197,198] of MMP-9 in mouse cerebral microvasculature, in retinal cells and in HUVECs. PEDF was demonstrated to suppress the expression and activities of MMP-2/9 in the retinas with severe neovascularization [88]. Interestingly, PEDF's regulatory effect on MMP-2/9 expression is also reported in a spontaneous pancreatic carcinoma model [52].

Plaminogen activator inhibitor-1 (PAI-1) is another angiogenic factor under the regulation of HIF-1, with hypoxia-response elements located at its promoter region [199–202]. PEDF suppresses PAI-1 transcription in HUVECs [203]. Interestingly, even under normal conditions, PEDF significantly decreases mRNA levels of PAI-1 in HUVECs [203]. Furthermore, the activity of plasma-derived PAI-1 is also substantially reduced by PEDF in rats [204].

### Common mechanisms for the anti-angiogenic activities of PEDF in diabetic and hypoxic conditions

**Figure 1:**
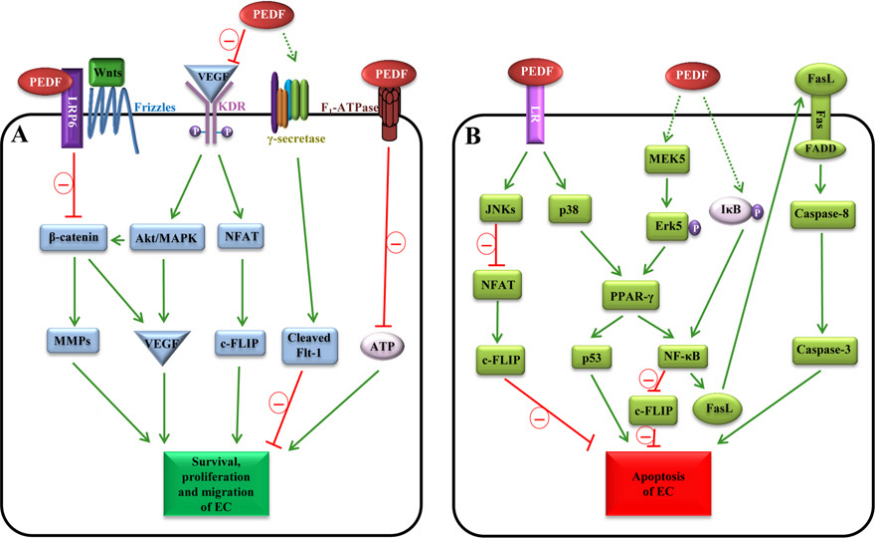
Molecular mechanisms for the anti-angiogenic activities of PEDF (**A**) PEDF blocks the survival, proliferation and migration of endothelial cells (ECs). PEDF binds to LRP6, an essential co-receptor of the Wnt/β-catenin pathway, which attenuates β-catenin nuclear translocation and subsequently the expression of angiogenic genes such as VEGF and MMPs. PEDF competes with VEGF for binding to KDR, inhibiting the downstream angiogenic Akt/MAPK and NFAT/c-FLIP pathways. The VEGF/Flt-1 signalling is critical for EC survival. PEDF elicits γ-secretase to cleave Flt-1, resulting in suppressed survival signalling of ECs. PEDF also binds to the β-subunit of F_1_-ATP synthase and inhibits the production of ATP, which is indispensable for EC angiogenic events. (**B**) PEDF promotes the apoptosis of ECs. PEDF activates JNKs through LR. Activated JNKs in the cytosol have higher binding affinity to NFATc2, resulting in cytosolic retention and thus less NFAT in the nucleus as a transcription factor that is required to promote the expression of an anti-apoptotic factor c-FLIP. PEDF also binds to LR to activate p38, which leads to activation of PPAR-γ. PEDF also activates PPAR-γ via the mediation of MEK5/Erk5. Activated PPAR-γ then stimulates p53 expression to induce EC apoptosis. NF-κB is another target gene of PPAR-γ. Increased NF-κB is able to up-regulate FasL expression to promote EC apoptosis via the Fas/FasL pathway. Moreover, NF-κB displaces NFAT and binds to the promoter of the c-FLIP gene, resulting in reduced levels of c-FLIP. Interestingly, PEDF also stimulates NF-κB via a PPAR-γ-independent pathway, i.e. by inducing IκB degradation. Red lines represent inhibition of pathways whereas continuous green arrows show activation of pathways. Broken green arrows illustrate activations of pathways whose detailed signalling cascades are currently not fully understood. Erk5, orphan MAPK; UCP-2, uncoupling protein 2.

In addition to the anti-angiogenic pathways mentioned above, a number of studies have demonstrated that under diabetic and hypoxic conditions, the common mechanisms by which PEDF inhibits angiogenesis are via promotion of endothelial cell death [9,124–126,159,192,205–214]. Both full-length PEDF [9,124,126,159,192,205–211] and PEDF-derived peptides [125,212–214] were reported to induce apoptosis of endothelial cells in *in vitro* culture or in angiogenic animal models. The apoptotic pathways in endothelial cells stimulated by PEDF are summarized as below (Figure 1).

The Fas/FasL interaction leads to programmed cell death [215]. Fas is constitutively expressed at low levels on the cell surface of quiescent endothelial cells. In angiogenic states, high concentrations of angiogenic stimulators increase cell surface presentation of Fas in activated endothelial cells [209,216]. PEDF induces the transcription and cell surface display of FasL in human dermal microvascular endothelial cells [209], leading to enhanced Fas/FasL interaction and subsequent activation of caspase 8-dependent apoptotic signalling. This action of PEDF on FasL has been suggested to be through activation of NF-κB [124]. In a bFGF-induced corneal neovascularization model, PEDF inhibits new vessel growth in wild-type mice, but not in FasL-deficient or Fas-deficient mice [209], further demonstrating that Fas/FasL mediates PEDF's anti-angiogenic effects. However, Fas/FasL/caspase-8 is not the only pathway to mediate PEDF's apoptotic effects in endothelial cells. It has been reported that deficiency of FasL or Fas does not attenuate the inhibitory effect of PEDF on hypoxia-induced angiogenesis in OIR retinas [217]. Moreover, a FasL-neutralizing antibody does not abolish PEDF-induced apoptosis in HUVECs [208], implying that alternative pathway(s) is(are) mediating PEDF's pro-apoptotic effects under these circumstances.

Another PEDF-elicited apoptotic pathway is the LR/JNK/NFAT/cellular FLICE-like inhibitory protein (c-FLIP)/caspase 8 signalling pathway. Both wild-type PEDF [126,192] and a triple phosphomimetic mutant of PEDF [126] activate JNKs in bovine aortic endothelial cells and HUVECs. The receptor that transmits PEDF's effect to JNKs is probably LR, as activation of JNKs is positively correlated with binding affinities of PEDF to LR, but not with those to PEDF-R [126]. Activated JNKs directly bind to NFATc2, resulting in cytoplasmic retention of NFATc2. Cytoplasmic retention of NFATc2 compromises the availability of NFAT in the nucleus as a transcription factor, which in turn leads to insufficient transcription of c-FLIP. As c-FLIP is an endogenous inhibitor of caspase 8, attenuated expression of c-FLIP results in enhanced activity of caspase 8 and subsequent endothelial cell apoptosis [192].

The LR/p38/PPAR-γ/apoptosis pathway also mediates PEDF's pro-apoptotic effects in endothelial cells. PEDF binds to LR [126] to activate p38 in bovine aortic endothelial cells and HUVECs [126,208,210,218]. Ho et al. [208,210] demonstrated that PEDF-stimulated p38 induces the expression and activity of PPAR-γ in HUVECs. Activation of PPAR-γ then induces p53 expression in HUVECs [208,210] to induce endothelial cell apoptosis [208,210]. In addition, PEDF also activates PPAR-γ via the MAPK/ERK kinase 5 (MEK5)/ERK5/PPAR-γ pathway [159]. PEDF induces Erk5 phosphorylation via MEK5, which then activates PPAR-γ [159]. PEDF-stimulated PPAR-γ not only induces the expression of p53 in HUVECs [208,210], but also promotes the expression and activity of NF-κB in human dermal capillary endothelial cells to suppress angiogenesis [159]. Interestingly, PPAR-γ-independent activation of NF-κB by PEDF was also reported: PEDF can induce the phosphorylation and degradation of inhibitor of κB kinase (IκB) in HUVECs [124]. As a result, activated NF-κB binds to the FasL promoter and initiates FasL transcription [124]. Moreover, PEDF-elicited NF-κB also displaces NFAT and binds to the promoter region of c-FLIP, resulting in decreased transcription of c-FLIP and enhanced endothelial cell apoptosis [124].

## REGULATION OF PEDF EXPRESSION, FUNCTION AND DEGRADATION

**Figure 2:**
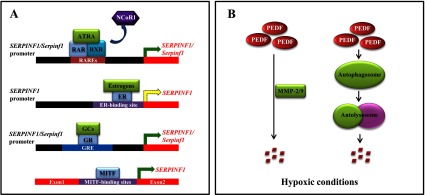
Regulation of PEDF expression, function and degradation (**A**) Transcriptional regulation of human *SERPINF1/*rodent *Serpinf1*. Six RAREs are located at −1000 to −1 bp of the *SERPINF1*/*Serpinf1* promoter region. In the presence of ATRA, RAR/RXR heterodimers bind to RAREs, dissociate from NCoR1, and recruit co-activators to activate *SERPINF1*/*Serpinf1* transcription. In the absence of agonists, RAR/RXR heterodimers associate with NCoR1, which suppresses *SERPINF1*/*Serpinf1* transcription. At least one ER-binding site is located at −864/+63 bp of the *SERPINF1* promoter. Oestrogens induce ERs to form either heterodimers or homodimers to bind to the ER-binding site, triggering/suppressing *SERPINF1* transcription depending on cell types and tissues. In addition, at least one GRE is found at −1721/+38 bp of the *SERPINF1*/*Serpinf1* promoter. GCs bind to GRs and promote GR nuclear translocation, which then initiates *SERPINF1*/*Serpinf1* transcription via the promoter region GRE. Three micropthalmia-associated transcription factor (MITF)-binding regions are identified within the first intron of the *SERPINF1* gene. MITF binds to the MITF-binding sites and up-regulates the transcription of *SERPINF1*. (**B**) Down-regulation of PEDF by hypoxia. Under hypoxic conditions, expression and activities of MMP-2/9 are increased, which promote the degradation of PEDF protein. In addition, a HIF-1-independent pathway to degrade PEDF was also reported. Hypoxia stimulates the autophagosome to down-regulate PEDF levels. Continuous green arrows represent gene transcription activation, whereas broken yellow arrows illustrate either activation or suppression of gene transcription depending on context. GCs, glucocorticoid/glucocorticoid analogues; GR, glucocorticoid receptor; GRE, glucocorticoid response element; NCoR1, nuclear receptor co-repressor 1.

PEDF levels decline in angiogenic tissues/organs [10,15–18,34,35]. In contrast, circulating PEDF levels increase in both Type 1 and Type 2 diabetes relative to patients without diabetes [24–27,44,45,47], which might be indicative of a systemic compensatory response to the decreased expression of PEDF in angiogenic tissues/organs. Understanding how PEDF levels are regulated may shed light on its roles in physiological and pathophysiological conditions. In the present review we discuss documented pathways that regulate PEDF levels (Figure 2), however, other mechanisms may also participate in the regulation of PEDF expression, function and degradation.

### Transcriptional regulation

Transcription of human *SERPINF1/*rodent *Serpinf1* genes is regulated by the binding of transcription factors to corresponding promoters. At −1000 to −1 bp of the human *SERPINF1* promoter region, six retinoic acid-response elements (RAREs) have been identified [219]. In the presence of all-*trans* retinoic acid (ATRA), heterodimers formed by retinoic acid receptors (RARs) and retinoid X receptors (RXRs) bind to RAREs, dissociate from co-repressors and recruit co-activators [219], leading to active transcription of the *SERPINF1*/*Serpinf1* gene. ATRA-induced expression of the *SERPINF1*/*Serpinf1* gene was reported in human retinal pigment epithelia and cancer cells [220,221], bovine retinal endothelial cells [220], mouse and rat neurons and neuron-derived cancer cells [220]. On the other hand, when agonists are absent, the nuclear receptor co-repressor 1 associates with the RAR/RXR heterodimer and recruits repressive complex to the RAREs, resulting in reduced transcription of the *SERPINF1* gene [219]. In addition, the *SERPINF1* promoter contains at least one oestrogen receptor (ER)-binding site located at −864/+63 bp [222,223]. ER has two isoforms that are recognized as ERα and ERβ. In the presence of agonists, ERs form either heterodimers or homodimers and then translocate into the nucleus, functioning as transcription factors. Oestrogen agonists, such as 17β-oestradiol and ginsenoside Rb1, significantly induce the transcription of *SERPINF1* in human Müller cells [224] and HUVECs [223]. 17β-Oestradiol-induced suppression of *SERPINF1* transcription was also reported in human ovarian epithelial cells [222], human endometrial cells [225] and rhesus retinal capillary endothelial cells [226]. Activation or repression of *SERPINF1* transcription probably depends on differential binding of ER heterodimers/homodimers to the promoter in different cell types or tissues. Corticosteroids and analogues, such as dexamethasone or triamcinolone, also regulate *SERPINF1*/*Serpinf1* expression via the glucocorticoid-response element located at the promoter region. It has been reported that there is at least one dexamethasone-binding site located at −1721/+38 bp of the human *SERPINF1* gene [227]. Up-regulation of *SERPINF1*/*Serpinf1* by corticosteriod analogues has been reported in a variety of cell types: HUVECs [228], primary human trabecular meshwork cells [229], human ARPE-19 cells [228], mouse 3T3-L1 cells [227], mouse Müller glial cells [220] and rat glioma cells [220]. Morever, three micropthalmia-associated transcription factor-binding regions are identified within the first intron of the human *SERPINF1* gene [230]. Up-regulation of PEDF by microthalmia-associated transcription factor is observed in human melanoma cell lines [230], primary melanocytes [230] and human RPE cells [231].

### Post-translational modification

Mature PEDF protein can undergo post-translational modifications such as N-terminal pyroglutamate blocking [232], phosphorylation [233,234] and glycosylation [81,232,235]. PEDF contains a conserved glycosylation motif N-X-L across species [5]. An N-linked glycosylation site is found in both human and bovine PEDF [81,232,235]. However, the role of N-linked glycosylation in PEDF function is yet to be investigated. It is reported that human PEDF protein could be phosphorylated by casein kinase 2 (CK2) and protein kinase A (PKA) [233]. CK2 phosphorylates PEDF at Ser^24^ and Ser^114^, whereas PKA phosphorylates Ser^227^ to a lesser extent [233]. The PEDF mutant S24E/S114E mimicking the CK2 phosphorylation exhibits enhanced anti-angiogenic effects, but decreased neurotrophic activities. In addition, the PEDF mutant S227E, which mimics the PKA phosphorylation, displays attenuated anti-angiogenic effects and intact neurotrophic activities [233,234]. PEDF isoforms with different molecular masses have been identified [83,235–237]. These isoforms display differential biological activities [235,236]. The machinery for PEDF isoform production and the mechanisms for the different activities of these isoforms of PEDF are presently unknown.

### Secretory regulation

The physiological concentration of PEDF in the human plasma is approximately 5 μg/ml (100 nM) [232]. The C-terminus (residues 415–418), RCL (residues 373–380), and hydrophobic core (Asn^391^–Thr^403^) corresponding to β-sheet B were found to be essential for human PEDF secretion [238]. The C-terminus truncated PEDF that lacks Arg^416^-Gly-Pro^418^ is secreted to a lesser extent compared with wild-type PEDF, and removal of Pro^415^-Arg-Gly-Pro^418^ completely abolishes PEDF secretion [238]. The transcription levels of these *SERPINF1* truncated mutants are similar to that of wild-type *SERPINF1*, but the PEDF mutant protein lacking Pro^415^ is only detected in the endoplasmic reticulum, whereas wild-type PEDF protein localizes to both the endoplasmic reticulum and the Golgi, suggesting that Pro^415^ at the C-terminus is essential for transporting PEDF protein from the endoplasmic reticulum to the Golgi [238]. Removal of RCL (∆373–380) also results in insufficient secretion due to impaired PEDF protein transport from the endoplasmic reticulum to the Golgi [238]. In addition, replacement of Gly^376^ and Leu^377^ with alanine within the RCL completely abolishes PEDF secretion, suggesting that the RCL plays an important role in the interaction of PEDF with the quality control mechinary within the endoplasmic reticulum [238]. Moreover, amino acid mutations of the hydrophobic core of β-sheet B results in no PEDF secretion, which is also due to compromised protein transport from the endoplasmic reticulum to the Golgi [238]. Interestingly, secretion of PEDF is predominantly apical in polarized RPE cells, which is an indicator of RPE polarization and full function [78–80]. Polarized and directional secretion of PEDF towards the neural retina by RPE cells results in abundant accumulation of PEDF in the interphotoreceptor matrix, and vitreous and aqueous humors [76,81–83]. The mechanism for polarized PEDF secretion in RPE cells is unknown.

### Degradative regulation

PEDF levels decrease in many disease conditions relative to non-disease conditions. Two degradative pathways regulating PEDF levels have been identified. Under hypoxia, the transcription factor HIF-1 is activated and induces the expression and activities of MMP-2/9 [193–198]. MMP-2/9 have been reported to proteolyze PEDF in a variety of cells including retinal cells [38,181,239,240]. A HIF-1-independent pathway to degrade PEDF was also reported, where hypoxia stimulated the autophagosome to down-regulate PEDF levels [241].

## POTENTIAL CLNICAL APPLICATIONS OF PEDF AS AN ANGIOGENIC INHIBITOR

On the basis of clinical correlations between circulating PEDF levels and diabetic angiogenesis, PEDF has been proposed as a biomarker for the assessment of angiogenesis progression in diabetic patients [24,27,44,47]. A Phase I clinical trial identified that PEDF had therapeutic effects in wet age-related macular degeneration, and the effect of PEDF as an anti-angiogenic agent in this model was promising [242]. Still, the potential of PEDF as an anti-angiogenic drug awaits further support from future clinical trials. In the present review, we provide a summary of possible strategies that may help explore the therapeutic potential of PEDF as an angiostatic factor.

Prior studies suggest that modification of PEDF protein may be a feasible therapeutic strategy. PEDF phosphomimetic mutants S24E/S114E [233] and S24E/S114E/S227E [234] exhibit enhanced anti-angiogenic effects compared with wild-type PEDF. The mature human PEDF protein (not including the 20-amino-acid signal peptide) contains 36 serine, 10 tyrosine and 29 threonine residues that can potentially be modified by phosphomimetics. Whether phosphomimetics at those sites will enhance the anti-angiogenic effects of PEDF in humans remains to be investigated. It was reported that binding to collagen I [134,135] potentiates PEDF's anti-angiogenic activities. PEDF is also capable of binding to collagen II [134], collagen III [136] and glycoaminoglycans [137–140]. Therefore modifications to enhance PEDF binding affinities to collagens and glycosoaminoglycans may be an additional strategy to improve PEDF's anti-angiogenic activity.

Development of PEDF isoforms or variants with enhanced anti-angiogenic properties is another alternative method. PEDF isoforms are found to exhibit different activities [235,236]. PEDF isoforms can result from different post-translational modifications, such as glycosylation by different oligosaccharides [232,235,243], N-terminal pyroglutamate blockage [232,243] and multiple phosphorylations [233,234]. Generation of PEDF isoforms via post-translational modifications provides another way to enhance the anti-angiogenic effects of PEDF.

Another strategy is to develop PEDF-derived peptides that possess potent anti-angiogenic activities. Peptide-based therapeutics are expected to be superior to full-length protein-based medicine in the following aspects: improved water solubility, higher production yield and purity by chemical synthesis, and lower immunogenicity [244]. Nevertheless, peptide-based medicines have their own disadvantages such as short half-lives. As summarized in Table 1, many functional PEDF-derived peptides possess anti-angiogenic activities, whereas the rest display biological activities that might be beneficial for the organism under diabetic and hypoxia-induced angiogenesis.

**Table 1:** Properties and amino acid positions of human PEDF-derived peptides in relation to angiogenesis Note: numbering of amino acids corresponds to the full-length human PEDF sequence, i.e. 418 amino acids including the 20-amino-acid signal peptide.

Properties	Positions and references	Cell or animal models used
Binding to PEDF receptor	Residues 44–77 [119,125,213,245], 44–121 [113], 46–70 [125], 78–121 [113,116,213]	Human Y-79, HuBMECs, T24 human urinary bladder carcinoma cells, HUVECs, human PC-3 cells and bovine retina plasma membrane
Binding to extracellular matrix	Residues 44–418 [134]	Not assayed
Neuron differentiation/neurotrophy	Residues 23–381 [6], 32–380 [6], 44–121 [6], 44–229 [6], 44–267 [6], 44–418 [246], 78–121 [113,213,247,248], 98–114 [213]	Human Y-79, embryonic rat motor neurons and human PC-3 cells
Neuroprotection	Residues 78–121 [154,247,249], 82–121 [250]	Human Y-79, embryonic rat motor neurons, diabetic retinal ganglion layer cells, rat organotypic spinal cord culture, mouse hypoxic retinal RGC layer, diabetic/ischaemic mouse inner plexiform layer and quinolinic acid-induced neurotoxicity in rat model
Anti-inflammation	Residues 60–77 [154], 78–121 [71,154], 82–121 [250]	Mouse retinal glial cells, mouse microglia, diabetic mouse vitreous and streptozotocin-induced diabetic mouse kidney
Anti-angiogenesis/anti-vasopermeability (anti-migration/anti-proliferation)	Residues 36–46 [213], 44–77 [91,212,213,251,252], 46–70 [125], 59–77 [212], 60–77 [154], 63–77 [212], 78–121 [154,253], 90–114 [62], 98–114 [213], 195–418 [254], 387–411 [62], 388–393 [93], 394–400 [93]	HuBMECs, b-FGF-induced corneal angiogenesis model, corneal micropocket assay, DIVAA, HUVECs, BRCEC, murine endothelial cells SVEC-4-10, mouse cornea, Akita mouse retina, matrigel plug assay on nude mouse, mouse corneal angiogenesis, PC-3 prostate cancer xenografts, Renca RCC exograft, VEGF-induced retinal vascular permeability mouse model, chicken embryo chorioallantoic membrane model OIR mouse model and laser-induced choroidal neovascularization rat model
Pro-apoptosis (in endothelial cells and tumour cells)	Residues 36–46 [213], 44–77 [212,213,245,251], 46–70 [125], 54–77 [212], 59–77 [212], 78–121 [213], 98–114 [213]	HuBMECs, bFGF-induced corneal angiogenesis model, BRCEC, human microvascular EC, T24 human urinary bladder carcinoma cells, HUVECs, mouse cornea, PC-3 cells, PC-3 prostate tumor xenograft and Renca RCC exograft

Overall, the anti-angiogenic effects and other beneficial properties of PEDF make it an attractive candidate as a clinical therapeutic agent for angiogenesis. However, PEDF-induced insulin resistance and inflammation remain as potential concerns for its therapeutic applications. A previous study by Crowe et al. [41] showed that acute PEDF treatment in lean mice produced compromised insulin sensitivity in the skeletal muscle and liver. More importantly, prolonged systemic PEDF administration resulted in diabetogenic effects, including increased lipolysis and subsequent ectopic lipid deposition in the skeletal muscle and liver [41]. In addition, some studies reported that PEDF promoted the expression of inflammatory factors and cytokines in rat microglia [255,256] and neonatal rat astrocytes [257]. Furthermore, the anti-angiogenic effects of PEDF are not always favourable in diabetic complications. For instance, elevated PEDF levels in the circulation of patients with Type 1 [27,44] and Type 2 diabetes [26,45,47,258] may lead to deficient peripheral angiogenesis and defective wound healing, which might worsen diabetic symptoms such as diabetic foot ulcers. This concept is supported by a recently published report [68] that increased plasma PEDF levels are detected in Type 2 diabetic patients with diabetic foot ulcers compared with diabetic patients without diabetic foot ulcer, and neutralizing PEDF in diabetic mice accelerates wound healing by increasing angiogenesis. It is currently unclear whether increased PEDF levels in the circulation play a pathogenic role, or are a compensatory response to angiogenic diseases. To circumvent the systemic side effects, direct administration of PEDF into the angiogenic tissues/organs for the treatment of proliferative DR or other angiogenic diseases is a potential delivery approach. Further efforts are needed to confirm the potential clinical application of PEDF.

## CONCLUSIONS

PEDF is a multifunctional serpin present in almost all tissues/organs and is involved in the maintenance of a variety of physiological functions. PEDF deficiency is known to play pathogenic roles in a number of diseased processes. Notably, PEDF levels are changed in diabetic and hypoxia-induced angiogenic diseases, which are believed to exacerbate the diseases. With broad activities and functions, PEDF has great clinical potential for disease diagnosis, treatment and prognosis prediction. However, its clinical application, especially its potential to combat pathological angiogenesis, remains to be explored.

## References

[1] Pignolo R.J., Cristofalo V.J., Rotenberg M.O. (1993). Senescent WI-38 cells fail to express EPC-1, a gene induced in young cells upon entry into the G0 state. J. Biol. Chem..

[2] Pignolo R.J., Rotenberg M.O., Cristofalo V.J. (1995). Analysis of EPC-1 growth state-dependent expression, specificity, and conservation of related sequences. J. Cell Physiol..

[3] Tombran-Tink J., Johnson L.V. (1989). Neuronal differentiation of retinoblastoma cells induced by medium conditioned by human RPE cells. Invest. Ophthalmol. Vis. Sci..

[4] Tombran-Tink J., Chader G.G., Johnson L.V. (1991). PEDF: a pigment epithelium-derived factor with potent neuronal differentiative activity. Exp. Eye Res..

[5] Tombran-Tink J., Aparicio S., Xu X., Tink A.R., Lara N., Sawant S., Barnstable C.J., Zhang S.S. (2005). PEDF and the serpins: phylogeny, sequence conservation, and functional domains. J. Struct. Biol..

[6] Becerra S.P., Sagasti A., Spinella P., Notario V. (1995). Pigment epithelium-derived factor behaves like a noninhibitory serpin. Neurotrophic activity does not require the serpin reactive loop. J. Biol. Chem..

[7] Becerra S.P. (1997). Structure-function studies on PEDF. A noninhibitory serpin with neurotrophic activity. Adv. Exp. Med. Biol..

[8] Dawson D.W., Volpert O.V., Gillis P., Crawford S.E., Xu H., Benedict W., Bouck N.P. (1999). Pigment epithelium-derived factor: a potent inhibitor of angiogenesis. Science.

[9] Stellmach V., Crawford S.E., Zhou W., Bouck N. (2001). Prevention of ischemia-induced retinopathy by the natural ocular antiangiogenic agent pigment epithelium-derived factor. Proc. Natl. Acad. Sci. U.S.A..

[10] Spranger J., Osterhoff M., Reimann M., Mohlig M., Ristow M., Francis M.K., Cristofalo V., Hammes H.P., Smith G., Boulton M., Pfeiffer A.F. (2001). Loss of the antiangiogenic pigment epithelium-derived factor in patients with angiogenic eye disease. Diabetes.

[11] Gao G., Li Y., Fant J., Crosson C.E., Becerra S.P., Ma J.X. (2002). Difference in ischemic regulation of vascular endothelial growth factor and pigment epithelium-derived factor in brown Norway and Sprague Dawley rats contributing to different susceptibilities to retinal neovascularization. Diabetes.

[12] Doll J.A., Stellmach V.M., Bouck N.P., Bergh A.R., Lee C., Abramson L.P., Cornwell M.L., Pins M.R., Borensztajn J., Crawford S.E. (2003). Pigment epithelium-derived factor regulates the vasculature and mass of the prostate and pancreas. Nat. Med..

[13] Crawford S.E., Stellmach V., Ranalli M., Huang X., Huang L., Volpert O., De Vries G.H., Abramson L.P., Bouck N. (2001). Pigment epithelium-derived factor (PEDF) in neuroblastoma: a multifunctional mediator of Schwann cell antitumor activity. J. Cell. Sci..

[14] Abramson L.P., Stellmach V., Doll J.A., Cornwell M., Arensman R.M., Crawford S.E. (2003). Wilms’ tumor growth is suppressed by antiangiogenic pigment epithelium-derived factor in a xenograft model. J. Pediatr. Surg..

[15] Yokoi M., Yamagishi S., Saito A., Yoshida Y., Matsui T., Saito W., Hirose S., Ohgami K., Kase M., Ohno S. (2007). Positive association of pigment epithelium-derived factor with total antioxidant capacity in the vitreous fluid of patients with proliferative diabetic retinopathy. Br. J. Ophthalmol..

[16] Boehm B.O., Lang G., Feldmann B., Kurkhaus A., Rosinger S., Volpert O., Lang G.K., Bouck N. (2003). Proliferative diabetic retinopathy is associated with a low level of the natural ocular anti-angiogenic agent pigment epithelium-derived factor (PEDF) in aqueous humor. a pilot study. Horm. Metab. Res..

[17] Boehm B.O., Lang G., Volpert O., Jehle P.M., Kurkhaus A., Rosinger S., Lang G.K., Bouck N. (2003). Low content of the natural ocular anti-angiogenic agent pigment epithelium-derived factor (PEDF) in aqueous humor predicts progression of diabetic retinopathy. Diabetologia.

[18] Ogata N., Nishikawa M., Nishimura T., Mitsuma Y., Matsumura M. (2002). Unbalanced vitreous levels of pigment epithelium-derived factor and vascular endothelial growth factor in diabetic retinopathy. Am. J. Ophthalmol..

[19] Guan M., Yam H.F., Su B., Chan K.P., Pang C.P., Liu W.W., Zhang W.Z., Lu Y. (2003). Loss of pigment epithelium derived factor expression in glioma progression. J. Clin. Pathol..

[20] Uehara H., Miyamoto M., Kato K., Ebihara Y., Kaneko H., Hashimoto H., Murakami Y., Hase R., Takahashi R., Mega S. (2004). Expression of pigment epithelium-derived factor decreases liver metastasis and correlates with favorable prognosis for patients with ductal pancreatic adenocarcinoma. Cancer Res..

[21] Zhang L., Chen J., Ke Y., Mansel R.E., Jiang W.G. (2006). Expression of pigment epithelial derived factor is reduced in non-small cell lung cancer and is linked to clinical outcome. Int. J. Mol. Med..

[22] Cai J., Parr C., Watkins G., Jiang W.G., Boulton M. (2006). Decreased pigment epithelium-derived factor expression in human breast cancer progression. Clin. Cancer Res..

[23] Holekamp N.M., Bouck N., Volpert O. (2002). Pigment epithelium-derived factor is deficient in the vitreous of patients with choroidal neovascularization due to age-related macular degeneration. Am. J. Ophthalmol..

[24] Matsuyama K., Ogata N., Matsuoka M., Shima C., Wada M., Jo N., Matsumura M. (2008). Relationship between pigment epithelium-derived factor (PEDF) and renal function in patients with diabetic retinopathy. Mol. Vis..

[25] Ogata N., Matsuoka M., Matsuyama K., Shima C., Tajika A., Nishiyama T., Wada M., Jo N., Higuchi A., Minamino K. (2007). Plasma concentration of pigment epithelium-derived factor in patients with diabetic retinopathy. J. Clin. Endocrinol. Metab..

[26] Chen H.B., Jia W.P., Lu J.X., Bao Y.Q., Li Q., Lu F.D., Lu W., Yu H.Y., Xiang K.S. (2007). Change and significance of serum pigment epithelium-derived factor in type 2 diabetic nephropathy. Zhonghua Yi Xue Za Zhi.

[27] Katakami N., Kaneto H., Yamasaki Y., Matsuhisa M. (2008). Increased serum pigment epithelium-derived factor levels in type 1 diabetic patients with diabetic retinopathy. Diabetes Res. Clin. Pract..

[28] Venturi G., Gandini A., Monti E., Dalle Carbonare L., Corradi M., Vincenzi M., Valenti M.T., Valli M., Pelilli E., Boner A. (2012). Lack of expression of SERPINF1, the gene coding for pigment epithelium-derived factor, causes progressively deforming osteogenesis imperfecta with normal type I collagen. J. Bone Miner. Res..

[29] Bogan R., Riddle R.C., Li Z., Kumar S., Nandal A., Faugere M.C., Boskey A., Crawford S.E., Clemens T.L. (2013). A mouse model for human osteogenesis imperfecta type VI. J. Bone Miner. Res..

[30] Huang Q., Wang S., Sorenson C.M., Sheibani N. (2008). PEDF-deficient mice exhibit an enhanced rate of retinal vascular expansion and are more sensitive to hyperoxia-mediated vessel obliteration. Exp. Eye Res..

[31] Chung C., Doll J.A., Gattu A.K., Shugrue C., Cornwell M., Fitchev P., Crawford S.E. (2008). Anti-angiogenic pigment epithelium-derived factor regulates hepatocyte triglyceride content through adipose triglyceride lipase (ATGL). J. Hepatol..

[32] Schmitz J.C., Protiva P., Gattu A.K., Utsumi T., Iwakiri Y., Neto A.G., Quinn M., Cornwell M.L., Fitchev P., Lugea A. (2011). Pigment epithelium-derived factor regulates early pancreatic fibrotic responses and suppresses the profibrotic cytokine thrombospondin-1. Am. J. Pathol..

[33] Shin E.S., Sorenson C.M., Sheibani N. (2013). PEDF expression regulates the pro-angiogenic and pro-inflammatory phenotype of the lung endothelium. Am. J. Physiol. Lung Cell. Mol. Physiol..

[34] Duh E.J., Yang H.S., Haller J.A., De Juan E., Humayun M.S., Gehlbach P., Melia M., Pieramici D., Harlan J.B., Campochiaro P.A., Zack D.J. (2004). Vitreous levels of pigment epithelium-derived factor and vascular endothelial growth factor: implications for ocular angiogenesis. Am. J. Ophthalmol..

[35] Yoshida Y., Yamagishi S., Matsui T., Nakamura K., Imaizumi T., Yoshimura K., Yamakawa R. (2007). Positive correlation of pigment epithelium-derived factor and total antioxidant capacity in aqueous humour of patients with uveitis and proliferative diabetic retinopathy. Br. J. Ophthalmol..

[36] Tombran-Tink J., Mazuruk K., Rodriguez I.R., Chung D., Linker T., Englander E., Chader G.J. (1996). Organization, evolutionary conservation, expression and unusual Alu density of the human gene for pigment epithelium-derived factor, a unique neurotrophic serpin. Mol. Vis..

[37] Becker J., Semler O., Gilissen C., Li Y., Bolz H.J., Giunta C., Bergmann C., Rohrbach M., Koerber F., Zimmermann K. (2011). Exome sequencing identifies truncating mutations in human SERPINF1 in autosomal-recessive osteogenesis imperfecta. Am. J. Hum. Genet..

[38] Chung C., Shugrue C., Nagar A., Doll J.A., Cornwell M., Gattu A., Kolodecik T., Pandol S.J., Gorelick F. (2009). Ethanol exposure depletes hepatic pigment epithelium-derived factor, a novel lipid regulator. Gastroenterology.

[39] Sawant S., Aparicio S., Tink A.R., Lara N., Barnstable C.J., Tombran-Tink J. (2004). Regulation of factors controlling angiogenesis in liver development: a role for PEDF in the formation and maintenance of normal vasculature. Biochem. Biophys. Res. Commun..

[40] Borg M.L., Andrews Z.B., Duh E.J., Zechner R., Meikle P.J., Watt M.J. (2011). Pigment epithelium-derived factor regulates lipid metabolism via adipose triglyceride lipase. Diabetes.

[41] Crowe S., Wu L.E., Economou C., Turpin S.M., Matzaris M., Hoehn K.L., Hevener A.L., James D.E., Duh E.J., Watt M.J. (2009). Pigment epithelium-derived factor contributes to insulin resistance in obesity. Cell Metab..

[42] Ho T.C., Chen S.L., Shih S.C., Wu J.Y., Han W.H., Cheng H.C., Yang S.L., Tsao Y.P. (2010). Pigment epithelium-derived factor is an intrinsic antifibrosis factor targeting hepatic stellate cells. Am. J. Pathol..

[43] Gattu A.K., Birkenfeld A.L., Jornayvaz F., Dziura J., Li F., Crawford S.E., Chu X., Still C.D., Gerhard G.S., Chung C., Samuel V. (2012). Insulin resistance is associated with elevated serum pigment epithelium-derived factor (PEDF) levels in morbidly obese patients. Acta Diabetol..

[44] Jenkins A.J., Zhang S.X., Rowley K.G., Karschimkus C.S., Nelson C.L., Chung J.S., O’Neal D.N., Januszewski A.S., Croft K.D., Mori T.A. (2007). Increased serum pigment epithelium-derived factor is associated with microvascular complications, vascular stiffness and inflammation in Type 1 diabetes. Diabet. Med..

[45] Jenkins A., Zhang S.X., Gosmanova A., Aston C., Dashti A., Baker M.Z., Lyons T., Ma J.X. (2008). Increased serum pigment epithelium derived factor levels in Type 2 diabetes patients. Diabetes Res. Clin. Pract..

[46] Nakamura K., Yamagishi S., Adachi H., Kurita-Nakamura Y., Matsui T., Inoue H. (2009). Serum levels of pigment epithelium-derived factor (PEDF) are positively associated with visceral adiposity in Japanese patients with type 2 diabetes. Diabetes Metab. Res. Rev..

[47] Jenkins A.J., Fu D., Azar M., Stoner J.A., Kaufman D.G., Zhang S., Klein R.L., Lopes-Virella M.F., Ma J.X., Lyons T.J., Vatd Investigators (2014). Clinical correlates of serum pigment epithelium-derived factor in type 2 diabetes patients. J. Diabetes Complications.

[48] Basu R., Chandramouli V., Dicke B., Landau B., Rizza R. (2005). Obesity and type 2 diabetes impair insulin-induced suppression of glycogenolysis as well as gluconeogenesis. Diabetes.

[49] Boden G., Cheung P., Homko C. (2003). Effects of acute insulin excess and deficiency on gluconeogenesis and glycogenolysis in type 1 diabetes. Diabetes.

[50] Torres T.P., Fujimoto Y., Donahue E.P., Printz R.L., Houseknecht K.L., Treadway J.L., Shiota M. (2011). Defective glycogenesis contributes toward the inability to suppress hepatic glucose production in response to hyperglycemia and hyperinsulinemia in zucker diabetic fatty rats. Diabetes.

[51] Samkharadze T., Erkan M., Reiser-Erkan C., Demir I.E., Kong B., Ceyhan G.O., Michalski C.W., Esposito I., Friess H., Kleeff J. (2011). Pigment epithelium-derived factor associates with neuropathy and fibrosis in pancreatic cancer. Am. J. Gastroenterol..

[52] Grippo P.J., Fitchev P.S., Bentrem D.J., Melstrom L.G., Dangi-Garimella S., Krantz S.B., Heiferman M.J., Chung C., Adrian K., Cornwell M.L. (2012). Concurrent PEDF deficiency and Kras mutation induce invasive pancreatic cancer and adipose-rich stroma in mice. Gut.

[53] Cosgrove G.P., Brown K.K., Schiemann W.P., Serls A.E., Parr J.E., Geraci M.W., Schwarz M.I., Cool C.D., Worthen G.S. (2004). Pigment epithelium-derived factor in idiopathic pulmonary fibrosis: a role in aberrant angiogenesis. Am. J. Respir. Crit. Care Med..

[54] Chetty A., Bennett M., Dang L., Nakamura D., Cao G.J., Mujahid S., Volpe M., Herman I., Becerra S.P., Nielsen H.C. (2014). Pigment epithelium-derived factor mediates impaired lung vascular development in neonatal hyperoxia. Am. J. Respir. Cell Mol. Biol..

[55] Hanumegowda C., Farkas L., Kolb M. (2012). Angiogenesis in pulmonary fibrosis: too much or not enough?. Chest.

[56] Quan G.M., Ojaimi J., Li Y., Kartsogiannis V., Zhou H., Choong P.F. (2005). Localization of pigment epithelium-derived factor in growing mouse bone. Calcif. Tissue Int..

[57] Tombran-Tink J., Barnstable C.J. (2004). Osteoblasts and osteoclasts express PEDF, VEGF-A isoforms, and VEGF receptors: possible mediators of angiogenesis and matrix remodeling in the bone. Biochem. Biophys. Res. Commun..

[58] Li F., Song N., Tombran-Tink J., Niyibizi C. (2014). Pigment epithelium derived factor suppresses expression of Sost/sclerostin by osteocytes: implication for its role in bone matrix mineralization. J. Cell Physiol..

[59] Akiyama T., Dass C.R., Shinoda Y., Kawano H., Tanaka S., Choong P.F. (2010). PEDF regulates osteoclasts via osteoprotegerin and RANKL. Biochem. Biophys. Res. Commun..

[60] Li F., Song N., Tombran-Tink J., Niyibizi C. (2013). Pigment epithelium derived factor enhances differentiation and mineral deposition of human mesenchymal stem cells. Stem Cells.

[61] Gattu A.K., Swenson E.S., Iwakiri Y., Samuel V.T., Troiano N., Berry R., Church C.D., Rodeheffer M.S., Carpenter T.O., Chung C. (2013). Determination of mesenchymal stem cell fate by pigment epithelium-derived factor (PEDF) results in increased adiposity and reduced bone mineral content. FASEB J..

[62] Ek E.T., Dass C.R., Contreras K.G., Choong P.F. (2007). PEDF-derived synthetic peptides exhibit antitumor activity in an orthotopic model of human osteosarcoma. J. Orthop. Res..

[63] Rauch F., Husseini A., Roughley P., Glorieux F.H., Moffatt P. (2012). Lack of circulating pigment epithelium-derived factor is a marker of osteogenesis imperfecta type VI. J. Clin. Endocrinol. Metab..

[64] Al-Jallad H., Palomo T., Moffatt P., Roughley P., Glorieux F.H., Rauch F. (2014). Normal bone density and fat mass in heterozygous SERPINF1 mutation carriers. J. Clin. Endocrinol. Metab..

[65] Farber C.R., Reich A., Barnes A.M., Becerra P., Rauch F., Cabral W.A., Bae A., Quinlan A., Glorieux F.H., Clemens T.L., Marini J.C. (2014). A novel IFITM5 mutation in severe atypical osteogenesis imperfecta type VI impairs osteoblast production of pigment epithelium-derived factor. J. Bone Miner. Res..

[66] Baron R., Kneissel M. (2013). WNT signaling in bone homeostasis and disease: from human mutations to treatments. Nat. Med..

[67] Park K., Lee K., Zhang B., Zhou T., He X., Gao G., Murray A.R., Ma J.X. (2011). Identification of a novel inhibitor of the canonical Wnt pathway. Mol. Cell Biol..

[68] Qi W., Yang C., Dai Z., Che D., Feng J., Mao Y., Cheng R., Wang Z., He X., Zhou T. (2014). High levels of pigment epithelium-derived factor in diabetes impair wound healing through suppression of Wnt signaling. Diabetes.

[69] Li X., Zhang Y., Kang H., Liu W., Liu P., Zhang J., Harris S.E., Wu D. (2005). Sclerostin binds to LRP5/6 and antagonizes canonical Wnt signaling. J. Biol. Chem..

[70] Abramson L.P., Browne M., Stellmach V., Doll J., Cornwell M., Reynolds M., Arensman R.M., Crawford S.E. (2006). Pigment epithelium-derived factor targets endothelial and epithelial cells in Wilms’ tumor. J. Pediatr. Surg..

[71] Awad A.S., Gao T., Gvritishvili A., You H., Liu Y., Cooper T.K., Reeves W.B., Tombran-Tink J. (2013). Protective role of small pigment epithelium-derived factor (PEDF) peptide in diabetic renal injury. Am. J. Physiol. Renal Physiol..

[72] Pina A.L., Kubitza M., Brawanski A., Tombran-Tink J., Kloth S. (2007). Expression of pigment-epithelium-derived factor during kidney development and aging. Cell Tissue Res..

[73] Wang J.J., Zhang S.X., Lu K., Chen Y., Mott R., Sato S., Ma J.X. (2005). Decreased expression of pigment epithelium-derived factor is involved in the pathogenesis of diabetic nephropathy. Diabetes.

[74] Shiga Y., Miura S., Mitsutake R., Yamagishi S., Saku K. (2011). Significance of plasma levels of pigment epithelium-derived factor as determined by multidetector row computed tomography in patients with mild chronic kidney disease and/or coronary artery disease. J. Int. Med. Res..

[75] Hui E., Yeung C.Y., Lee P.C., Woo Y.C., Fong C.H., Chow W.S., Xu A., Lam K.S. (2014). Elevated circulating pigment epithelium-derived factor predicts the progression of diabetic nephropathy in patients with type 2 diabetes. J. Clin. Endocrinol. Metab..

[76] Karakousis P.C., John S.K., Behling K.C., Surace E.M., Smith J.E., Hendrickson A., Tang W.X., Bennett J., Milam A.H. (2001). Localization of pigment epithelium derived factor (PEDF) in developing and adult human ocular tissues. Mol. Vis..

[77] Kozulin P., Natoli R., Bumsted O’Brien K.M., Madigan M.C., Provis J.M. (2010). The cellular expression of antiangiogenic factors in fetal primate macula. Invest. Ophthalmol. Vis. Sci..

[78] Becerra S.P., Fariss R.N., Wu Y.Q., Montuenga L.M., Wong P., Pfeffer B.A. (2004). Pigment epithelium-derived factor in the monkey retinal pigment epithelium and interphotoreceptor matrix: apical secretion and distribution. Exp. Eye Res..

[79] Sonoda S., Sreekumar P.G., Kase S., Spee C., Ryan S.J., Kannan R., Hinton D.R. (2010). Attainment of polarity promotes growth factor secretion by retinal pigment epithelial cells: relevance to age-related macular degeneration. Aging (Albany NY).

[80] Zhu D., Deng X., Spee C., Sonoda S., Hsieh C.L., Barron E., Pera M., Hinton D.R. (2011). Polarized secretion of PEDF from human embryonic stem cell-derived RPE promotes retinal progenitor cell survival. Invest. Ophthalmol. Vis. Sci..

[81] Wu Y.Q., Notario V., Chader G.J., Becerra S.P. (1995). Identification of pigment epithelium-derived factor in the interphotoreceptor matrix of bovine eyes. Protein Expr. Purif..

[82] Wu Y.Q., Becerra S.P. (1996). Proteolytic activity directed toward pigment epithelium-derived factor in vitreous of bovine eyes. Implications of proteolytic processing. Invest. Ophthalmol. Vis. Sci..

[83] Tombran-Tink J., Shivaram S.M., Chader G.J., Johnson L.V., Bok D. (1995). Expression, secretion, and age-related downregulation of pigment epithelium-derived factor, a serpin with neurotrophic activity. J. Neurosci..

[84] Behling K.C., Surace E.M., Bennett J. (2002). Pigment epithelium-derived factor expression in the developing mouse eye. Mol. Vis..

[85] Ogata N., Wada M., Otsuji T., Jo N., Tombran-Tink J., Matsumura M. (2002). Expression of pigment epithelium-derived factor in normal adult rat eye and experimental choroidal neovascularization. Invest. Ophthalmol. Vis. Sci..

[86] Sorenson C.M., Wang S., Gendron R., Paradis H., Sheibani N. (2013). Thrombospondin-1 deficiency exacerbates the pathogenesis of diabetic retinopathy. J. Diabetes Metab.

[87] Park K., Jin J., Hu Y., Zhou K., Ma J.X. (2011). Overexpression of pigment epithelium-derived factor inhibits retinal inflammation and neovascularization. Am. J. Pathol..

[88] Haurigot V., Villacampa P., Ribera A., Bosch A., Ramos D., Ruberte J., Bosch F. (2012). Long-term retinal PEDF overexpression prevents neovascularization in a murine adult model of retinopathy. PLoS ONE.

[89] Mori K., Duh E., Gehlbach P., Ando A., Takahashi K., Pearlman J., Mori K., Yang H.S., Zack D.J., Ettyreddy D. (2001). Pigment epithelium-derived factor inhibits retinal and choroidal neovascularization. J. Cell Physiol..

[90] Saishin Y., Silva R.L., Saishin Y., Kachi S., Aslam S., Gong Y.Y., Lai H., Carrion M., Harris B., Hamilton M. (2005). Periocular gene transfer of pigment epithelium-derived factor inhibits choroidal neovascularization in a human-sized eye. Hum. Gene Ther..

[91] Amaral J., Becerra S.P. (2010). Effects of human recombinant PEDF protein and PEDF-derived peptide 34-mer on choroidal neovascularization. Invest. Ophthalmol. Vis. Sci..

[92] Kuo C.N., Chen C.Y., Chen S.N., Yang L.C., Lai L.J., Lai C.H., Chen M.F., Hung C.H., Chen C.H. (2013). Inhibition of corneal neovascularization with the combination of bevacizumab and plasmid pigment epithelium-derived factor-synthetic amphiphile interaction-18 (p-PEDF-SAINT-18) vector in a rat corneal experimental angiogenesis model. Int. J. Mol. Sci..

[93] Matsui T., Nishino Y., Maeda S., Yamagishi S. (2012). PEDF-derived peptide inhibits corneal angiogenesis by suppressing VEGF expression. Microvasc. Res..

[94] Kuo C.N., Yang L.C., Yang C.T., Lai C.H., Chen M.F., Chen C.Y., Chen C.H., Wu P.C., Kou H.K., Chen Y.J. (2009). Inhibition of corneal neovascularization with plasmid pigment epithelium-derived factor (p-PEDF) delivered by synthetic amphiphile interaction-18 (SAINT-18) vector in an experimental model of rat corneal angiogenesis. Exp. Eye Res..

[95] Volpert K.N., Tombran-Tink J., Barnstable C., Layer P.G. (2009). PEDF and GDNF are key regulators of photoreceptor development and retinal neurogenesis in reaggregates from chick embryonic retina. J. Ocul. Biol. Dis. Infor..

[96] Malchiodi-Albedi F., Feher J., Caiazza S., Formisano G., Perilli R., Falchi M., Petrucci T.C., Scorcia G., Tombran-Tink J. (1998). PEDF (pigment epithelium-derived factor) promotes increase and maturation of pigment granules in pigment epithelial cells in neonatal albino rat retinal cultures. Int. J. Dev. Neurosci..

[97] Arnhold S., Heiduschka P., Klein H., Absenger Y., Basnaoglu S., Kreppel F., Henke-Fahle S., Kochanek S., Bartz-Schmidt K.U., Addicks K., Schraermeyer U. (2006). Adenovirally transduced bone marrow stromal cells differentiate into pigment epithelial cells and induce rescue effects in RCS rats. Invest. Ophthalmol. Vis. Sci..

[98] Cayouette M., Smith S.B., Becerra S.P., Gravel C. (1999). Pigment epithelium-derived factor delays the death of photoreceptors in mouse models of inherited retinal degenerations. Neurobiol. Dis..

[99] Cao W., Tombran-Tink J., Chen W., Mrazek D., Elias R., McGinnis J.F. (1999). Pigment epithelium-derived factor protects cultured retinal neurons against hydrogen peroxide-induced cell death. J. Neurosci. Res..

[100] Wang Y., Subramanian P., Shen D., Tuo J., Becerra S.P., Chan C.C. (2013). Pigment epithelium-derived factor reduces apoptosis and pro-inflammatory cytokine gene expression in a murine model of focal retinal degeneration. ASN NEURO.

[101] Cao W., Tombran-Tink J., Elias R., Sezate S., Mrazek D., McGinnis J.F. (2001). *In vivo* protection of photoreceptors from light damage by pigment epithelium-derived factor. Invest. Ophthalmol. Vis. Sci..

[102] Imai D., Yoneya S., Gehlbach P.L., Wei L.L., Mori K. (2005). Intraocular gene transfer of pigment epithelium-derived factor rescues photoreceptors from light-induced cell death. J. Cell Physiol..

[103] Jablonski M.M., Tombran-Tink J., Mrazek D.A., Iannaccone A. (2000). Pigment epithelium-derived factor supports normal development of photoreceptor neurons and opsin expression after retinal pigment epithelium removal. J. Neurosci..

[104] Subramanian P., Locatelli-Hoops S., Kenealey J., DesJardin J., Notari L., Becerra S.P. (2013). Pigment epithelium-derived factor (PEDF) prevents retinal cell death via PEDF receptor (PEDF-R): identification of a functional ligand binding site. J. Biol. Chem..

[105] Folkman J., Klagsbrun M. (1987). Angiogenic factors. Science.

[106] Wietecha M.S., Cerny W.L., DiPietro L.A. (2013). Mechanisms of vessel regression: toward an understanding of the resolution of angiogenesis. Curr. Top Microbiol. Immunol..

[107] Hoshina D., Abe R., Yamagishi S.I., Shimizu H. (2010). The role of PEDF in tumor growth and metastasis. Curr. Mol. Med..

[108] Manalo K.B., Choong P.F., Becerra S.P., Dass C.R. (2011). Pigment epithelium-derived factor as an anticancer drug and new treatment methods following the discovery of its receptors: a patent perspective. Expert. Opin. Ther. Pat..

[109] Becerra S.P., Notario V. (2013). The effects of PEDF on cancer biology: mechanisms of action and therapeutic potential. Nat. Rev. Cancer.

[110] Rychli K., Huber K., Wojta J. (2009). Pigment epithelium-derived factor (PEDF) as a therapeutic target in cardiovascular disease. Expert Opin. Ther. Targets..

[111] Liu J.T., Chen Y.L., Chen W.C., Chen H.Y., Lin Y.W., Wang S.H., Man K.M., Wan H.M., Yin W.H., Liu P.L., Chen Y.H. (2012). Role of pigment epithelium-derived factor in stem/progenitor cell-associated neovascularization. J. Biomed. Biotechnol..

[112] Notari L., Baladron V., Aroca-Aguilar J.D., Balko N., Heredia R., Meyer C., Notario P.M., Saravanamuthu S., Nueda M.L., Sanchez-Sanchez F. (2006). Identification of a lipase-linked cell membrane receptor for pigment epithelium-derived factor. J. Biol. Chem..

[113] Alberdi E., Aymerich M.S., Becerra S.P. (1999). Binding of pigment epithelium-derived factor (PEDF) to retinoblastoma cells and cerebellar granule neurons. Evidence for a PEDF receptor. J. Biol. Chem..

[114] Subramanian P., Notario P.M., Becerra S.P. (2010). Pigment epithelium-derived factor receptor (PEDF-R): a plasma membrane-linked phospholipase with PEDF binding affinity. Adv. Exp. Med. Biol..

[115] Desjardin J.T., Becerra S.P., Subramanian P. (2013). Searching for alternatively spliced variants of phospholipase domain-containing 2 (Pnpla2), a novel gene in the retina. J. Clin. Exp. Ophthalmol..

[116] Aymerich M.S., Alberdi E.M., Martinez A., Becerra S.P. (2001). Evidence for pigment epithelium-derived factor receptors in the neural retina. Invest. Ophthalmol. Vis. Sci..

[117] Subramanian P., Rapp M., Becerra S.P. (2012). Identification of pigment epithelium-derived factor receptor (PEDF-R) antibody epitopes. Adv. Exp. Med. Biol..

[118] Hirsch J., Johnson C.L., Nelius T., Kennedy R., Riese W., Filleur S. (2011). PEDF inhibits IL8 production in prostate cancer cells through PEDF receptor/phospholipase A2 and regulation of NFkappaB and PPARgamma. Cytokine.

[119] Ladhani O., Sanchez-Martinez C., Orgaz J.L., Jimenez B., Volpert O.V. (2011). Pigment epithelium-derived factor blocks tumor extravasation by suppressing amoeboid morphology and mesenchymal proteolysis. Neoplasia.

[120] Chavan S.S., Hudson L.K., Li J.H., Ochani M., Harris Y., Patel N.B., Katz D., Scheinerman J.A., Pavlov V.A., Tracey K.J. (2012). Identification of pigment epithelium-derived factor as an adipocyte-derived inflammatory factor. Mol. Med..

[121] Moreno-Navarrete J.M., Touskova V., Sabater M., Mraz M., Drapalova J., Ortega F., Serrano M., Catalan V., Gomez-Ambrosi J., Ortiz M.R. (2013). Liver, but not adipose tissue PEDF gene expression is associated with insulin resistance. Int. J. Obes. (Lond)..

[122] Gonzalez R., Jennings L.L., Knuth M., Orth A.P., Klock H.E., Ou W., Feuerhelm J., Hull M.V., Koesema E., Wang Y. (2010). Screening the mammalian extracellular proteome for regulators of embryonic human stem cell pluripotency. Proc. Natl. Acad. Sci. U.S.A..

[123] Rapp M., Woo G., Al-Ubaidi M.R., Becerra S.P., Subramanian P. (2014). Pigment epithelium-derived factor protects cone photoreceptor-derived 661W cells from light damage through Akt activation. Adv. Exp. Med. Biol..

[124] Aurora A.B., Biyashev D., Mirochnik Y., Zaichuk T.A., Sanchez-Martinez C., Renault M.A., Losordo D., Volpert O.V. (2010). NF-kappaB balances vascular regression and angiogenesis via chromatin remodeling and NFAT displacement. Blood.

[125] Bernard A., Gao-Li J., Franco C.A., Bouceba T., Huet A., Li Z. (2009). Laminin receptor involvement in the anti-angiogenic activity of pigment epithelium-derived factor. J. Biol. Chem..

[126] Konson A., Pradeep S., D’Acunto C.W., Seger R. (2011). Pigment epithelium-derived factor and its phosphomimetic mutant induce JNK-dependent apoptosis and p38-mediated migration arrest. J. Biol. Chem..

[127] Notari L., Arakaki N., Mueller D., Meier S., Amaral J., Becerra S.P. (2010). Pigment epithelium-derived factor binds to cell-surface F(1)-ATP synthase. FEBS J..

[128] Willnow T.E., Goldstein J.L., Orth K., Brown M.S., Herz J. (1992). Low density lipoprotein receptor-related protein and gp330 bind similar ligands, including plasminogen activator-inhibitor complexes and lactoferrin, an inhibitor of chylomicron remnant clearance. J. Biol. Chem..

[129] Conese M., Olson D., Blasi F. (1994). Protease nexin-1-urokinase complexes are internalized and degraded through a mechanism that requires both urokinase receptor and alpha 2-macroglobulin receptor. J. Biol. Chem..

[130] Poller W., Willnow T.E., Hilpert J., Herz J. (1995). Differential recognition of alpha 1-antitrypsin-elastase and alpha 1-antichymotrypsin-cathepsin G complexes by the low density lipoprotein receptor-related protein. J. Biol. Chem..

[131] Zhang B., Zhou K.K., Ma J.X. (2010). Inhibition of connective tissue growth factor overexpression in diabetic retinopathy by SERPINA3K via blocking the WNT/beta-catenin pathway. Diabetes.

[132] Hu Y., Chen Y., Lin M., Lee K., Mott R.A., Ma J.X. (2013). Pathogenic role of the Wnt signaling pathway activation in laser-induced choroidal neovascularization. Invest. Ophthalmol. Vis. Sci..

[133] Lee K., Hu Y., Ding L., Chen Y., Takahashi Y., Mott R., Ma J.X. (2012). Therapeutic potential of a monoclonal antibody blocking the Wnt pathway in diabetic retinopathy. Diabetes.

[134] Meyer C., Notari L., Becerra S.P. (2002). Mapping the type I collagen-binding site on pigment epithelium-derived factor. Implications for its antiangiogenic activity. J. Biol. Chem..

[135] Hosomichi J., Yasui N., Koide T., Soma K., Morita I. (2005). Involvement of the collagen I-binding motif in the anti-angiogenic activity of pigment epithelium-derived factor. Biochem. Biophys. Res. Commun..

[136] Kozaki K., Miyaishi O., Koiwai O., Yasui Y., Kashiwai A., Nishikawa Y., Shimizu S., Saga S. (1998). Isolation, purification, and characterization of a collagen-associated serpin, caspin, produced by murine colon adenocarcinoma cells. J. Biol. Chem..

[137] Alberdi E., Hyde C.C., Becerra S.P. (1998). Pigment epithelium-derived factor (PEDF) binds to glycosaminoglycans: analysis of the binding site. Biochemistry.

[138] Becerra S.P., Perez-Mediavilla L.A., Weldon J.E., Locatelli-Hoops S., Senanayake P., Notari L., Notario V., Hollyfield J.G. (2008). Pigment epithelium-derived factor binds to hyaluronan. Mapping of a hyaluronan binding site. J. Biol. Chem..

[139] Valnickova Z., Petersen S.V., Nielsen S.B., Otzen D.E., Enghild J.J. (2007). Heparin binding induces a conformational change in pigment epithelium-derived factor. J. Biol. Chem..

[140] Alberdi E.M., Weldon J.E., Becerra S.P. (2003). Glycosaminoglycans in human retinoblastoma cells: heparan sulfate, a modulator of the pigment epithelium-derived factor-receptor interactions. BMC Biochem..

[141] Martin A., Komada M.R., Sane D.C. (2003). Abnormal angiogenesis in diabetes mellitus. Med. Res. Rev..

[142] Nyengaard J.R., Rasch R. (1993). The impact of experimental diabetes mellitus in rats on glomerular capillary number and sizes. Diabetologia.

[143] Mohan N., Monickaraj F., Balasubramanyam M., Rema M., Mohan V. (2012). Imbalanced levels of angiogenic and angiostatic factors in vitreous, plasma and postmortem retinal tissue of patients with proliferative diabetic retinopathy. J. Diabetes Complications.

[144] Wang H., Feng L., Hu J.W., Xie C.L., Wang F. (2012). Characterisation of the vitreous proteome in proliferative diabetic retinopathy. Proteome. Sci..

[145] Huber M., Wachtlin J. (2012). Vitreous levels of proteins implicated in angiogenesis are modulated in patients with retinal or choroidal neovascularization. Ophthalmologica.

[146] Zheng B., Li T., Chen H., Xu X., Zheng Z. (2011). Correlation between ficolin-3 and vascular endothelial growth factor-to-pigment epithelium-derived factor ratio in the vitreous of eyes with proliferative diabetic retinopathy. Am. J. Ophthalmol..

[147] Matsunaga N., Chikaraishi Y., Izuta H., Ogata N., Shimazawa M., Matsumura M., Hara H. (2008). Role of soluble vascular endothelial growth factor receptor-1 in the vitreous in proliferative diabetic retinopathy. Ophthalmology.

[148] Murugeswari P., Shukla D., Rajendran A., Kim R., Namperumalsamy P., Muthukkaruppan V. (2008). Proinflammatory cytokines and angiogenic and anti-angiogenic factors in vitreous of patients with proliferative diabetic retinopathy and eales’ disease. Retina.

[149] Garcia-Ramirez M., Canals F., Hernandez C., Colome N., Ferrer C., Carrasco E., Garcia-Arumi J., Simo R. (2007). Proteomic analysis of human vitreous fluid by fluorescence-based difference gel electrophoresis (DIGE): a new strategy for identifying potential candidates in the pathogenesis of proliferative diabetic retinopathy. Diabetologia.

[150] Wang J.J., Zhang S.X., Mott R., Knapp R.R., Cao W., Lau K., Ma J.X. (2006). Salutary effect of pigment epithelium-derived factor in diabetic nephropathy: evidence for antifibrogenic activities. Diabetes.

[151] Arimura T., Miura S., Sugihara M., Iwata A., Yamagishi S., Saku K. (2011). Association between plasma levels of pigment epithelium-derived factor and renal dysfunction in patients with coronary artery disease. Cardiol. J..

[152] Yoshida Y., Yamagishi S., Matsui T., Jinnouchi Y., Fukami K., Imaizumi T., Yamakawa R. (2009). Protective role of pigment epithelium-derived factor (PEDF) in early phase of experimental diabetic retinopathy. Diabetes Metab. Res. Rev..

[153] Zhang S.X., Wang J.J., Gao G., Parke K., Ma J.X. (2006). Pigment epithelium-derived factor downregulates vascular endothelial growth factor (VEGF) expression and inhibits VEGF-VEGF receptor 2 binding in diabetic retinopathy. J. Mol. Endocrinol..

[154] Liu Y., Leo L.F., McGregor C., Grivitishvili A., Barnstable C.J., Tombran-Tink J. (2012). Pigment epithelium-derived factor (PEDF) peptide eye drops reduce inflammation, cell death and vascular leakage in diabetic retinopathy in Ins2(Akita) mice. Mol. Med..

[155] Wang J.J., Zhang S.X., Mott R., Chen Y., Knapp R.R., Cao W., Ma J.X. (2008). Anti-inflammatory effects of pigment epithelium-derived factor in diabetic nephropathy. Am. J. Physiol. Renal Physiol..

[156] Rundhaug J.E. (2005). Matrix metalloproteinases and angiogenesis. J. Cell. Mol. Med..

[157] Yafai Y., Lange J., Wiedemann P., Reichenbach A., Eichler W. (2007). Pigment epithelium-derived factor acts as an opponent of growth-stimulatory factors in retinal glial-endothelial cell interactions. Glia.

[158] Hutchings H., Maitre-Boube M., Tombran-Tink J., Plouet J. (2002). Pigment epithelium-derived factor exerts opposite effects on endothelial cells of different phenotypes. Biochem. Biophys. Res. Commun..

[159] Biyashev D., Veliceasa D., Kwiatek A., Sutanto M.M., Cohen R.N., Volpert O.V. (2010). Natural angiogenesis inhibitor signals through Erk5 activation of peroxisome proliferator-activated receptor gamma (PPARgamma). J. Biol. Chem..

[160] Dejana E. (2010). The role of Wnt signaling in physiological and pathological angiogenesis. Circ. Res..

[161] Zhang X., Gaspard J.P., Chung D.C. (2001). Regulation of vascular endothelial growth factor by the Wnt and K-ras pathways in colonic neoplasia. Cancer Res..

[162] Brabletz T., Jung A., Dag S., Hlubek F., Kirchner T. (1999). β-Catenin regulates the expression of the matrix metalloproteinase-7 in human colorectal cancer. Am. J. Pathol..

[163] Crawford H.C., Fingleton B.M., Rudolph-Owen L.A., Goss K.J., Rubinfeld B., Polakis P., Matrisian L.M. (1999). The metalloproteinase matrilysin is a target of β-catenin transactivation in intestinal tumors. Oncogene.

[164] Wu B., Crampton S.P., Hughes C.C. (2007). Wnt signaling induces matrix metalloproteinase expression and regulates T cell transmigration. Immunity.

[165] Yamagishi S., Nakamura K., Matsui T., Inagaki Y., Takenaka K., Jinnouchi Y., Yoshida Y., Matsuura T., Narama I., Motomiya Y. (2006). Pigment epithelium-derived factor inhibits advanced glycation end product-induced retinal vascular hyperpermeability by blocking reactive oxygen species-mediated vascular endothelial growth factor expression. J. Biol. Chem..

[166] Yamagishi S., Matsui T., Nakamura K., Yoshida T., Takeuchi M., Inoue H., Yoshida Y., Imaizumi T. (2007). Pigment-epithelium-derived factor suppresses expression of receptor for advanced glycation end products in the eye of diabetic rats. Ophthalmic Res..

[167] Yamagishi S., Inagaki Y., Nakamura K., Abe R., Shimizu T., Yoshimura A., Imaizumi T. (2004). Pigment epithelium-derived factor inhibits TNF-alpha-induced interleukin-6 expression in endothelial cells by suppressing NADPH oxidase-mediated reactive oxygen species generation. J. Mol. Cell Cardiol..

[168] Yamagishi S., Nakamura K., Ueda S., Kato S., Imaizumi T. (2005). Pigment epithelium-derived factor (PEDF) blocks angiotensin II signaling in endothelial cells via suppression of NADPH oxidase: a novel anti-oxidative mechanism of PEDF. Cell Tissue Res..

[169] Zheng Z., Chen H., Zhao H., Liu K., Luo D., Chen Y., Chen Y., Yang X., Gu Q., Xu X. (2010). Inhibition of JAK2/STAT3-mediated VEGF upregulation under high glucose conditions by PEDF through a mitochondrial ROS pathway *in vitro*. Invest. Ophthalmol. Vis. Sci..

[170] Ejaz S., Chekarova I., Ejaz A., Sohail A., Lim C.W. (2008). Importance of pericytes and mechanisms of pericyte loss during diabetes retinopathy. Diabetes Obes. Metab..

[171] Zhang S.X., Wang J.J., Dashti A., Wilson K., Zou M.H., Szweda L., Ma J.X., Lyons T.J. (2008). Pigment epithelium-derived factor mitigates inflammation and oxidative stress in retinal pericytes exposed to oxidized low-density lipoprotein. J. Mol. Endocrinol..

[172] Yamagishi S., Inagaki Y., Amano S., Okamoto T., Takeuchi M., Makita Z. (2002). Pigment epithelium-derived factor protects cultured retinal pericytes from advanced glycation end product-induced injury through its antioxidative properties. Biochem. Biophys. Res. Commun..

[173] Amano S., Yamagishi S., Inagaki Y., Nakamura K., Takeuchi M., Inoue H., Imaizumi T. (2005). Pigment epithelium-derived factor inhibits oxidative stress-induced apoptosis and dysfunction of cultured retinal pericytes. Microvasc. Res..

[174] Enge M., Bjarnegard M., Gerhardt H., Gustafsson E., Kalen M., Asker N., Hammes H.P., Shani M., Fassler R., Betsholtz C. (2002). Endothelium-specific platelet-derived growth factor-B ablation mimics diabetic retinopathy. EMBO J..

[175] Yamagishi S., Nakamura K., Takenaka K., Matsui T., Jinnouchi Y., Imaizumi T. (2005). Pigment epithelium-derived factor (PEDF) promotes growth of pericytes through autocrine production of platelet-derived growth factor-B. Microvasc. Res..

[176] Wang G.L., Jiang B.H., Rue E.A., Semenza G.L. (1995). Hypoxia-inducible factor 1 is a basic-helix-loop-helix-PAS heterodimer regulated by cellular O_2_ tension. Proc. Natl. Acad. Sci. U.S.A..

[177] Pugh C.W., Ratcliffe P.J. (2003). Regulation of angiogenesis by hypoxia: role of the HIF system. Nat. Med..

[178] Ke Q., Costa M. (2006). Hypoxia-inducible factor-1 (HIF-1). Mol Pharmacol..

[179] Smith L.E., Wesolowski E., McLellan A., Kostyk S.K., D’Amato R., Sullivan R., D’Amore P.A. (1994). Oxygen-induced retinopathy in the mouse. Invest. Ophthalmol. Vis. Sci..

[180] Mowat F.M., Luhmann U.F., Smith A.J., Lange C., Duran Y., Harten S., Shukla D., Maxwell P.H., Ali R.R., Bainbridge J.W. (2010). HIF-1alpha and HIF-2alpha are differentially activated in distinct cell populations in retinal ischaemia. PLoS ONE.

[181] Notari L., Miller A., Martinez A., Amaral J., Ju M., Robinson G., Smith L.E., Becerra S.P. (2005). Pigment epithelium-derived factor is a substrate for matrix metalloproteinase type 2 and type 9: implications for downregulation in hypoxia. Invest. Ophthalmol. Vis. Sci..

[182] Miller H., Miller B., Ishibashi T., Ryan S.J. (1990). Pathogenesis of laser-induced choroidal subretinal neovascularization. Invest. Ophthalmol. Vis. Sci..

[183] Mori K., Gehlbach P., Yamamoto S., Duh E., Zack D.J., Li Q., Berns K.I., Raisler B.J., Hauswirth W.W., Campochiaro P.A. (2002). AAV-mediated gene transfer of pigment epithelium-derived factor inhibits choroidal neovascularization. Invest. Ophthalmol. Vis. Sci..

[184] Hamilton M.M., Byrnes G.A., Gall J.G., Brough D.E., King C.R., Wei L.L. (2008). Alternate serotype adenovector provides long-term therapeutic gene expression in the eye. Mol. Vis..

[185] Liu Y., Cox S.R., Morita T., Kourembanas S. (1995). Hypoxia regulates vascular endothelial growth factor gene expression in endothelial cells. Identification of a 5′ enhancer. Circ. Res..

[186] Shweiki D., Itin A., Soffer D., Keshet E. (1992). Vascular endothelial growth factor induced by hypoxia may mediate hypoxia-initiated angiogenesis. Nature.

[187] Zhang Z., Neiva K.G., Lingen M.W., Ellis L.M., Nor J.E. (2010). VEGF-dependent tumor angiogenesis requires inverse and reciprocal regulation of VEGFR1 and VEGFR2. Cell Death Differ..

[188] Cai J., Jiang W.G., Grant M.B., Boulton M. (2006). Pigment epithelium-derived factor inhibits angiogenesis via regulated intracellular proteolysis of vascular endothelial growth factor receptor 1. J. Biol. Chem..

[189] Kendall R.L., Thomas K.A. (1993). Inhibition of vascular endothelial cell growth factor activity by an endogenously encoded soluble receptor. Proc. Natl. Acad. Sci. U.S.A..

[190] Zou D., Zhang Z., He J., Zhang K., Ye D., Han W., Zhou J., Wang Y., Li Q., Liu X. (2012). Blood vessel formation in the tissue-engineered bone with the constitutively active form of HIF-1alpha mediated BMSCs. Biomaterials.

[191] Calvani M., Rapisarda A., Uranchimeg B., Shoemaker R.H., Melillo G. (2006). Hypoxic induction of an HIF-1alpha-dependent bFGF autocrine loop drives angiogenesis in human endothelial cells. Blood.

[192] Zaichuk T.A., Shroff E.H., Emmanuel R., Filleur S., Nelius T., Volpert O.V. (2004). Nuclear factor of activated T cells balances angiogenesis activation and inhibition. J. Exp. Med..

[193] Ben-Yosef Y., Lahat N., Shapiro S., Bitterman H., Miller A. (2002). Regulation of endothelial matrix metalloproteinase-2 by hypoxia/reoxygenation. Circ. Res..

[194] Rodrigues M., Xin X., Jee K., Babapoor-Farrokhran S., Kashiwabuchi F., Ma T., Bhutto I., Hassan S.J., Daoud Y., Baranano D. (2013). VEGF secreted by hypoxic Muller cells induces MMP-2 expression and activity in endothelial cells to promote retinal neovascularization in proliferative diabetic retinopathy. Diabetes.

[195] Cheng X.W., Kuzuya M., Kim W., Song H., Hu L., Inoue A., Nakamura K., Di Q., Sasaki T., Tsuzuki M. (2010). Exercise training stimulates ischemia-induced neovascularization via phosphatidylinositol 3-kinase/Akt-dependent hypoxia-induced factor-1 alpha reactivation in mice of advanced age. Circulation.

[196] Yamamoto Y., Osanai T., Nishizaki F., Sukekawa T., Izumiyama K., Sagara S., Okumura K. (2012). Matrix metalloprotein-9 activation under cell-to-cell interaction between endothelial cells and monocytes: possible role of hypoxia and tumor necrosis factor-alpha. Heart Vessels.

[197] Bauer A.T., Burgers H.F., Rabie T., Marti H.H. (2010). Matrix metalloproteinase-9 mediates hypoxia-induced vascular leakage in the brain via tight junction rearrangement. J. Cereb. Blood Flow Metab..

[198] Hollborn M., Stathopoulos C., Steffen A., Wiedemann P., Kohen L., Bringmann A. (2007). Positive feedback regulation between MMP-9 and VEGF in human RPE cells. Invest. Ophthalmol. Vis. Sci..

[199] Kaluz S., Kaluzova M., Stanbridge E.J. (2008). Rational design of minimal hypoxia-inducible enhancers. Biochem. Biophys. Res. Commun..

[200] Patel N., Sundaram N., Yang M., Madigan C., Kalra V.K., Malik P. (2010). Placenta growth factor (PlGF), a novel inducer of plasminogen activator inhibitor-1 (PAI-1) in sickle cell disease (SCD). J. Biol. Chem..

[201] Liao H., Hyman M.C., Lawrence D.A., Pinsky D.J. (2007). Molecular regulation of the PAI-1 gene by hypoxia: contributions of Egr-1, HIF-1alpha, and C/EBPalpha. FASEB J..

[202] Jung S.Y., Song H.S., Park S.Y., Chung S.H., Kim Y.J. (2011). Pyruvate promotes tumor angiogenesis through HIF-1-dependent PAI-1 expression. Int. J. Oncol..

[203] Matsui T., Higashimoto Y., Taira J., Yamagishi S. (2013). Pigment epithelium-derived factor (PEDF) binds to caveolin-1 and inhibits the pro-inflammatory effects of caveolin-1 in endothelial cells. Biochem. Biophys. Res. Commun..

[204] Yamagishi S., Matsui T., Nakamura K., Takenaka K. (2009). Administration of pigment epithelium-derived factor prolongs bleeding time by suppressing plasminogen activator inhibitor-1 activity and platelet aggregation in rats. Clin. Exp. Med..

[205] Bai Y.J., Huang L.Z., Xu X.L., Du W., Zhou A.Y., Yu W.Z., Li X.X. (2012). Polyethylene glycol-modified pigment epithelial-derived factor: new prospects for treatment of retinal neovascularization. J. Pharmacol. Exp. Ther..

[206] Konson A., Pradeep S., Seger R. (2010). Phosphomimetic mutants of pigment epithelium-derived factor with enhanced antiangiogenic activity as potent anticancer agents. Cancer Res..

[207] Yang J., Chen S., Huang X., Han J., Wang Q., Shi D., Cheng R., Gao G., Yang X. (2010). Growth suppression of cervical carcinoma by pigment epithelium-derived factor via anti-angiogenesis. Cancer Biol. Ther..

[208] Ho T.C., Chen S.L., Yang Y.C., Liao C.L., Cheng H.C., Tsao Y.P. (2007). PEDF induces p53-mediated apoptosis through PPAR gamma signaling in human umbilical vein endothelial cells. Cardiovasc. Res..

[209] Volpert O.V., Zaichuk T., Zhou W., Reiher F., Ferguson T.A., Stuart P.M., Amin M., Bouck N.P. (2002). Inducer-stimulated Fas targets activated endothelium for destruction by anti-angiogenic thrombospondin-1 and pigment epithelium-derived factor. Nat. Med..

[210] Ho T.C., Chen S.L., Yang Y.C., Lo T.H., Hsieh J.W., Cheng H.C., Tsao Y.P. (2009). Cytosolic phospholipase A2-α is an early apoptotic activator in PEDF-induced endothelial cell apoptosis. Am. J. Physiol. Cell Physiol..

[211] Mori K., Gehlbach P., Ando A., McVey D., Wei L., Campochiaro P.A. (2002). Regression of ocular neovascularization in response to increased expression of pigment epithelium-derived factor. Invest. Ophthalmol. Vis. Sci..

[212] Mirochnik Y., Aurora A., Schulze-Hoepfner F.T., Deabes A., Shifrin V., Beckmann R., Polsky C., Volpert O.V. (2009). Short pigment epithelial-derived factor-derived peptide inhibits angiogenesis and tumor growth. Clin. Cancer Res..

[213] Filleur S., Volz K., Nelius T., Mirochnik Y., Huang H., Zaichuk T.A., Aymerich M.S., Becerra S.P., Yap R., Veliceasa D. (2005). Two functional epitopes of pigment epithelial-derived factor block angiogenesis and induce differentiation in prostate cancer. Cancer Res..

[214] Gong Q., Yang X., Cai W., Gao G., Yang Z. (2010). Expression and purification of functional epitope of pigment epithelium-derived factor in *E. coli* with inhibiting effect on endothelial cells. Protein J..

[215] Ju S.T., Panka D.J., Cui H., Ettinger R., el-Khatib M., Sherr D.H., Stanger B.Z., Marshak-Rothstein A. (1995). Fas(CD95)/FasL interactions required for programmed cell death after T-cell activation. Nature.

[216] Kaplan H.J., Leibole M.A., Tezel T., Ferguson T.A. (1999). Fas ligand (CD95 ligand) controls angiogenesis beneath the retina. Nat. Med..

[217] Barreiro R., Schadlu R., Herndon J., Kaplan H.J., Ferguson T.A. (2003). The role of Fas-FasL in the development and treatment of ischemic retinopathy. Invest. Ophthalmol. Vis. Sci..

[218] Chen L., Zhang S.S., Barnstable C.J., Tombran-Tink J. (2006). PEDF induces apoptosis in human endothelial cells by activating p38 MAP kinase dependent cleavage of multiple caspases. Biochem. Biophys. Res. Commun..

[219] Doyon G., St-Jean S., Darsigny M., Asselin C., Boudreau F. (2009). Nuclear receptor co-repressor is required to maintain proliferation of normal intestinal epithelial cells in culture and down-modulates the expression of pigment epithelium-derived factor. J. Biol. Chem..

[220] Tombran-Tink J., Lara N., Apricio S.E., Potluri P., Gee S., Ma J.X., Chader G., Barnstable C.J. (2004). Retinoic acid and dexamethasone regulate the expression of PEDF in retinal and endothelial cells. Exp. Eye Res..

[221] Uchida H., Hayashi H., Kuroki M., Uno K., Yamada H., Yamashita Y., Tombran-Tink J., Kuroki M., Oshima K. (2005). Vitamin A up-regulates the expression of thrombospondin-1 and pigment epithelium-derived factor in retinal pigment epithelial cells. Exp. Eye Res..

[222] Cheung L.W., Au S.C., Cheung A.N., Ngan H.Y., Tombran-Tink J., Auersperg N., Wong A.S. (2006). Pigment epithelium-derived factor is estrogen sensitive and inhibits the growth of human ovarian cancer and ovarian surface epithelial cells. Endocrinology.

[223] Leung K.W., Cheung L.W., Pon Y.L., Wong R.N., Mak N.K., Fan T.P., Au S.C., Tombran-Tink J., Wong A.S. (2007). Ginsenoside Rb1 inhibits tube-like structure formation of endothelial cells by regulating pigment epithelium-derived factor through the oestrogen beta receptor. Br. J. Pharmacol..

[224] Li C., Tang Y., Li F., Turner S., Li K., Zhou X., Centola M., Yan X., Cao W. (2006). 17β-Estradiol (betaE2) protects human retinal Muller cell against oxidative stress *in vitro*: evaluation of its effects on gene expression by cDNA microarray. Glia.

[225] Chuderland D., Ben-Ami I., Friedler S., Hasky N., Ninio-Many L., Goldberg K., Bar-Joseph H., Grossman H., Shalgi R. (2014). Hormonal regulation of pigment epithelium-derived factor (PEDF) expression in the endometrium. Mol. Cell Endocrinol..

[226] Parvathaneni K., Grigsby J.G., Betts B.S., Tsin A.T. (2013). Estrogen-induced retinal endothelial cell proliferation: possible involvement of pigment epithelium-derived factor and phosphoinositide 3-kinase/mitogen-activated protein kinase pathways. J. Ocul. Pharmacol. Ther..

[227] Zhou Y., Xu F., Deng H., Bi Y., Sun W., Zhao Y., Chen Z., Weng J. (2013). PEDF expression is inhibited by insulin treatment in adipose tissue via suppressing 11beta-HSD1. PLoS ONE.

[228] Tong J.P., Lam D.S., Chan W.M., Choy K.W., Chan K.P., Pang C.P. (2006). Effects of triamcinolone on the expression of VEGF and PEDF in human retinal pigment epithelial and human umbilical vein endothelial cells. Mol. Vis..

[229] Perruccio E.M., Rowlette L.L., Comes N., Locatelli-Hoops S., Notari L., Becerra S.P., Borras T. (2008). Dexamethasone increases pigment epithelium-derived factor in perfused human eyes. Curr. Eye Res..

[230] Fernandez-Barral A., Orgaz J.L., Baquero P., Ali Z., Moreno A., Tiana M., Gomez V., Riveiro-Falkenbach E., Canadas C., Zazo S. (2014). Regulatory and functional connection of microphthalmia-associated transcription factor and anti-metastatic pigment epithelium derived factor in melanoma. Neoplasia.

[231] Ma X., Pan L., Jin X., Dai X., Li H., Wen B., Chen Y., Ma A., Qu J., Hou L. (2012). Microphthalmia-associated transcription factor acts through PEDF to regulate RPE cell migration. Exp. Cell Res..

[232] Petersen S.V., Valnickova Z., Enghild J.J. (2003). Pigment-epithelium-derived factor (PEDF) occurs at a physiologically relevant concentration in human blood: purification and characterization. Biochem. J..

[233] Maik-Rachline G., Shaltiel S., Seger R. (2005). Extracellular phosphorylation converts pigment epithelium-derived factor from a neurotrophic to an antiangiogenic factor. Blood.

[234] Maik-Rachline G., Seger R. (2006). Variable phosphorylation states of pigment-epithelium-derived factor differentially regulate its function. Blood.

[235] Duh E.J., Yang H.S., Suzuma I., Miyagi M., Youngman E., Mori K., Katai M., Yan L., Suzuma K., West K. (2002). Pigment epithelium-derived factor suppresses ischemia-induced retinal neovascularization and VEGF-induced migration and growth. Invest. Ophthalmol. Vis. Sci..

[236] Subramanian P., Deshpande M., Locatelli-Hoops S., Moghaddam-Taaheri S., Gutierrez D., Fitzgerald D.P., Guerrier S., Rapp M., Notario V., Becerra S.P. (2012). Identification of pigment epithelium-derived factor protein forms with distinct activities on tumor cell lines. J. Biomed. Biotechnol..

[237] Wang S., Gottlieb J.L., Sorenson C.M., Sheibani N. (2009). Modulation of thrombospondin 1 and pigment epithelium-derived factor levels in vitreous fluid of patients with diabetes. Arch. Ophthalmol..

[238] Shao H., Schvartz I., Shaltiel S. (2003). Secretion of pigment epithelium-derived factor. Mutagenic study. Eur. J. Biochem..

[239] Xiao Q., Zeng S., Lv M., Ling S. (2008). Small hairpin loop RNA targeting HIF-1alpha down-regulates VEGF and up-regulates PEDF in human retinal pigment epithelial cells under hypoxic condition. J. Huazhong. Univ. Sci. Technolog. Med. Sci..

[240] Bandyopadhyay M., Rohrer B. (2012). Matrix metalloproteinase activity creates pro-angiogenic environment in primary human retinal pigment epithelial cells exposed to complement. Invest. Ophthalmol. Vis. Sci..

[241] Fernandez-Barral A., Orgaz J.L., Gomez V., del Peso L., Calzada M.J., Jimenez B. (2012). Hypoxia negatively regulates antimetastatic PEDF in melanoma cells by a hypoxia inducible factor-independent, autophagy dependent mechanism. PLoS ONE.

[242] Rasmussen H., Chu K.W., Campochiaro P., Gehlbach P.L., Haller J.A., Handa J.T., Nguyen Q.D., Sung J.U. (2001). Clinical protocol. An open-label, phase I, single administration, dose-escalation study of ADGVPEDF.11D (ADPEDF) in neovascular age-related macular degeneration (AMD). Hum. Gene. Ther..

[243] Conti A., Ricchiuto P., Iannaccone S., Sferrazza B., Cattaneo A., Bachi A., Reggiani A., Beltramo M., Alessio M. (2005). Pigment epithelium-derived factor is differentially expressed in peripheral neuropathies. Proteomics.

[244] Craik D.J., Fairlie D.P., Liras S., Price D. (2013). The future of peptide-based drugs. Chem. Biol. Drug Des..

[245] Deshpande M., Notari L., Subramanian P., Notario V., Becerra S.P. (2012). Inhibition of tumor cell surface ATP synthesis by pigment epithelium-derived factor: implications for antitumor activity. Int. J. Oncol..

[246] Becerra S.P., Palmer I., Kumar A., Steele F., Shiloach J., Notario V., Chader G.J. (1993). Overexpression of fetal human pigment epithelium-derived factor in *Escherichia coli*. A functionally active neurotrophic factor. J. Biol. Chem..

[247] Bilak M.M., Becerra S.P., Vincent A.M., Moss B.H., Aymerich M.S., Kuncl R.W. (2002). Identification of the neuroprotective molecular region of pigment epithelium-derived factor and its binding sites on motor neurons. J. Neurosci..

[248] Smith N.D., Schulze-Hoepfner F.T., Veliceasa D., Filleur S., Shareef S., Huang L., Huang X.M., Volpert O.V. (2008). Pigment epithelium-derived factor and interleukin-6 control prostate neuroendocrine differentiation via feed-forward mechanism. J. Urol..

[249] Sanagi T., Yabe T., Yamada H. (2010). Adenoviral gene delivery of pigment epithelium-derived factor protects striatal neurons from quinolinic acid-induced excitotoxicity. J. Neuropathol. Exp. Neurol..

[250] Li H., Tran V.V., Hu Y., Mark Saltzman W., Barnstable C.J., Tombran-Tink J. (2006). A PEDF N-terminal peptide protects the retina from ischemic injury when delivered in PLGA nanospheres. Exp. Eye Res..

[251] Longeras R., Farjo K., Ihnat M., Ma J.X. (2012). A PEDF-derived peptide inhibits retinal neovascularization and blocks mobilization of bone marrow-derived endothelial progenitor cells. Exp. Diabetes Res..

[252] Esipov R.S., Beirakhova K.A., Chupova L.A., Likhvantseva V.K., Stepanova E.V., Miroshnikov A.I. (2012). Recombinant fragment of pigment epithelium-derived factor (44–77) prevents pathological corneal neovascularization. Bioorg. Khim..

[253] Liu H., Ren J.G., Cooper W.L., Hawkins C.E., Cowan M.R., Tong P.Y. (2004). Identification of the antivasopermeability effect of pigment epithelium-derived factor and its active site. Proc. Natl. Acad. Sci. U.S.A..

[254] Sanchez-Sanchez F., Aroca-Aguilar J.D., Segura I., Ramirez-Castillejo C., Riese H.H., Coca-Prados M., Escribano J. (2008). Expression and purification of functional recombinant human pigment epithelium-derived factor (PEDF) secreted by the yeast *Pichia pastoris*. J. Biotechnol..

[255] Sanagi T., Yabe T., Yamada H. (2005). The regulation of pro-inflammatory gene expression induced by pigment epithelium-derived factor in rat cultured microglial cells. Neurosci. Lett..

[256] Takanohashi A., Yabe T., Schwartz J.P. (2005). Pigment epithelium-derived factor induces the production of chemokines by rat microglia. Glia.

[257] Yabe T., Sanagi T., Schwartz J.P., Yamada H. (2005). Pigment epithelium-derived factor induces pro-inflammatory genes in neonatal astrocytes through activation of NF-kappa B and CREB. Glia.

[258] Li S., Fu X.A., Zhou X.F., Chen Y.Y., Chen W.Q. (2012). Angiogenesis-related cytokines in serum of proliferative diabetic retinopathy patients before and after vitrectomy. Int. J. Ophthalmol..

